# ReactELISA method for quantifying methylglyoxal levels in plasma and cell cultures

**DOI:** 10.1016/j.redox.2019.101252

**Published:** 2019-06-11

**Authors:** Rasmus Kold-Christensen, Karina Kragh Jensen, Emil Smedegård-Holmquist, Lambert Kristiansen Sørensen, Jakob Hansen, Karl Anker Jørgensen, Peter Kristensen, Mogens Johannsen

**Affiliations:** a Department of Forensic Medicine, Aarhus University, Palle Juul-Jensens Boulevard 99, 8200 Aarhus N, Denmark; b Department of Chemistry, Aarhus University, Langelandsgade 140, 8000 Aarhus C, Denmark; c Department of Engineering, Aarhus University, Gustav Wieds Vej 10, 8000 Aarhus C, Denmark

**Keywords:** Methylglyoxal, ELISA, Glyoxalase, Plasma, Cell culture, Buthionine sulfoximine

## Abstract

Methylglyoxal (MG) is a toxic glycolytic by-product associated with increased levels of inflammation and oxidative stress and has been linked to ageing-related diseases, such as diabetes and Alzheimer's disease. As MG is a highly reactive dicarbonyl compound, forming both reversible and irreversible adducts with a range of endogenous nucleophiles, measuring endogenous levels of MG are quite troublesome. Furthermore, as MG is a small metabolite it is not very immunogenic, excluding conventional ELISA for detection purposes, thus only more instrumentally demanding LC-MS/MS-based methods have demonstrated convincing quantitative data. In the present work we develop a novel bifunctional MG capture probe as well as a high specificity monoclonal antibody to finally setup a robust reaction-based ELISA (ReactELISA) method for detecting the highly reactive and low-level (nM) metabolite MG in human biological specimens. The assay is tested and validated against the current golden standard LC-MS/MS method in human blood plasma and cell-culture media. Furthermore, we demonstrate the assays ability to measure small perturbations of MG levels in growth media caused by a small molecule drug buthionine sulfoximine (BSO) of current clinical relevance. Finally, the assay is converted into a homogenous (no-wash) AlphaLISA version (ReactAlphaLISA), which offers the potential for operationally simple screening of further small molecules capable of perturbing cellular MG. Such compounds could be of relevance as probes to gain insight into MG metabolism as well as drug-leads to alleviate ageing-related diseases.

## Highlights

• MG is challenging to quantify, here we present a simple and specific ReactELISA based approach and validate against LC-MS/MS.
• Sensitivity at low (nM) endogenous concentration in both human blood plasma and cell culture media.
• Impact of BSO treatment of HEK293 cells can be profiled in culture media.
• Potential use in cell-based phenotypic screen for small molecules modulating MG metabolism.

## Graphical abstract

**Figure:**
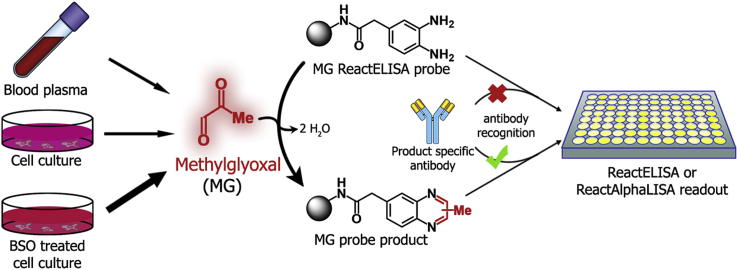

## 1 Introduction

Methylglyoxal (MG) is a highly reactive α-oxoaldehyde metabolite ubiquitous in all living organisms [1]. It is mainly produced as a toxic by-product from glycolysis, but to a lesser extent also originates from other pathways, such as lipid and protein metabolism, as well as non-enzymatic degradation of monosaccharides [[2], [3], [4], [5]]. Elevated levels of MG has been linked to a vast amount of different ageing-related pathologies, including Alzheimer's and Parkinson's disease [6], diabetic complications [7], oxidative stress [8], and reduced longevity in simple organisms [9]. The toxicity of MG is primarily due to its ability to form advanced glycation end-products (AGEs); non-enzymatic post-translational protein modifications that may disrupt normal protein function [10,11]. To prevent MG from forming AGEs, most organisms have a glyoxalase system, which in humans consist of two cytosolic enzymes; glyoxalase 1 (GLO1) and glyoxalase 2 (GLO2) [12]. The glyoxalase system is the primary pathway responsible for the detoxification of MG by converting it into non-toxic d-lactate using reduced gluthatione (GSH) as a cofactor [13,14]. In support of the glyoxalase system is the aldo-keto reductases (AKR) which have been shown to metabolize MG into hydroxyacetone independently of GSH [15]. While the glyoxalase system is the major detoxification pathway, AKR activity may also be physiologically relevant as increased AKR activity e.g. have been linked to reduced diabetic complications [16]. Normal human endogenous levels of MG is in the range of 50–300 nM in blood plasma and 1000–2000 nM intracellularly depending on tissue type [17]. Higher levels of MG is observed for example, in patients with diabetic neuropathy, where plasma concentrations have been reported to be as high as 600–900 nM [7]. However, as more than 99% of the MG in cells is estimated to be reversibly bound to thiols, primarily GSH, and a range of sources for MG exist, the correct measurement of MG is a continuous debate and challenge [13,17,18].

Due to the importance of MG and its derived AGEs in relation to human pathologies, fast, easy, and reliable methods for quantifying MG is required. To our knowledge, no data on measurements of unbound MG in biological specimens as e.g. plasma or growth media have been reported in the literature using ordinary antibody or enzyme-based methods. The closest proxy reported being ELISA-measurements of specific MG-protein adducts (AGEs) reflecting historical MG levels [19]. Robust and accurate quantification has only been achieved by derivatization of MG using reagents such as *o*-phenylenediamine, followed by detection using chromatography and mass spectrometry [17,20]. While these techniques are both accurate and precise, the sample through-put is limited and the analysis requires expensive specialized equipment and training limiting more general use [21]. Other recent more operationally simple methods, such as a gold nanoparticle-based technique suffers from a detection limit being 1 μM MG in pre-treated and diluted plasma [22]. Another method is a “Turn-On” fluorescent sensor for MG [23]. The fluorogenic “Turn On” probe has been capable of quantifying MG in plasma with high (~650 nM)  MG concentrations, though it more adequately is used for fluorescence microscopy imaging.

**Fig. 1:**
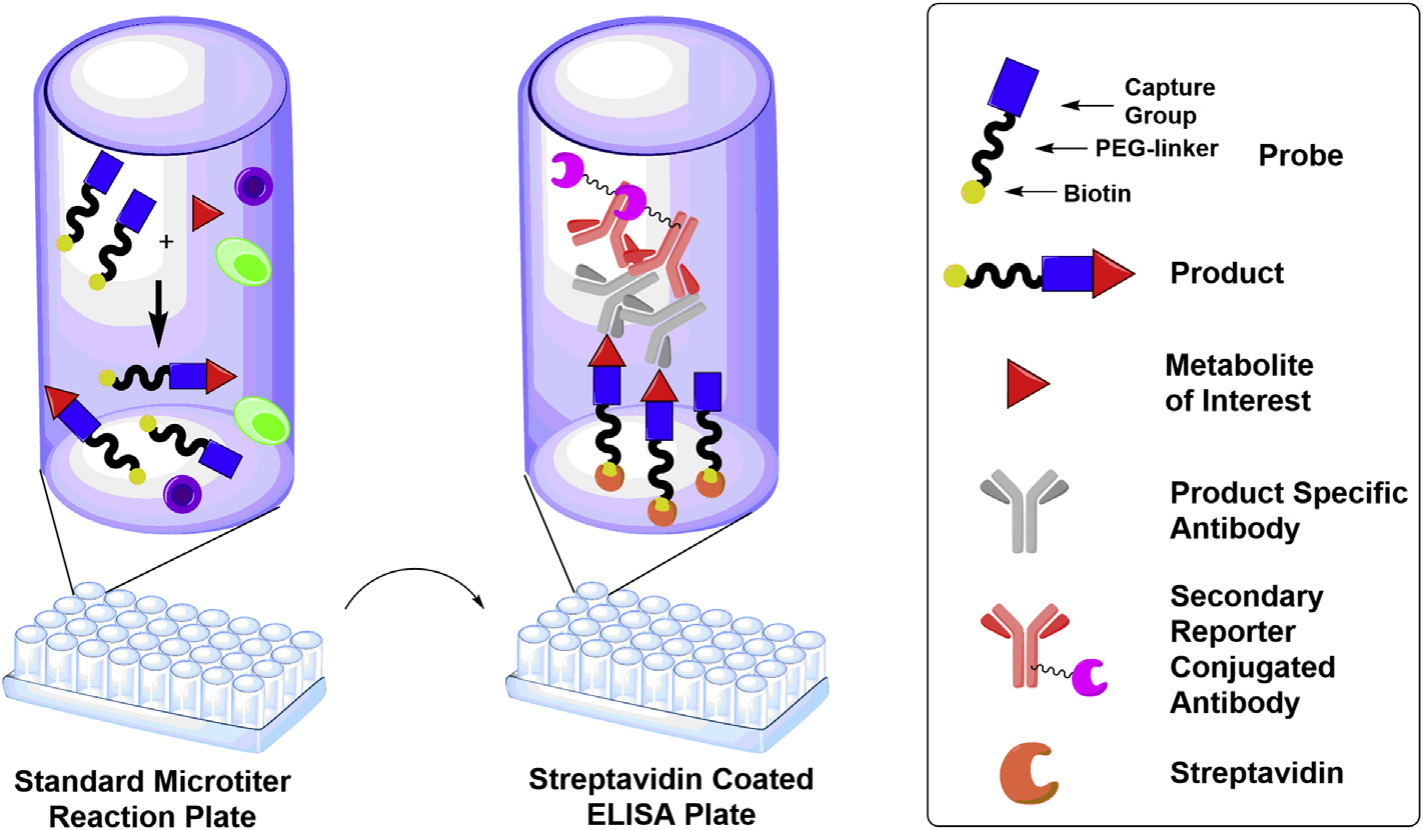
The principle of ReactELISA. The bifunctional ReactELISA probe is mixed with a biological matrix containing the reactive metabolite of interest e.g. methylglyoxal and allowed to react in a microtiter well. After the reaction, the probe/product mixture is immobilized in streptavidin-coated wells allowing purification by washing away unbound matrix. The product is then quantified by ELISA using a product-specific antibody.

To overcome these limitations and obtain a tool for operationally simple MG measurements, we here report on the development of a novel bifunctional biotin MG capture probe as well as a highly specific product antibody and their application in ReactELISA (Fig. 1) as well as homogenous ReactAlphaLISA (Fig. 5) assays to quantitate low nM levels of MG in biological samples [24].

## 2 Materials and methods

For a detailed description of all protocols, procedures, and syntheses see the Supporting Information.

### 2.1 Sample preparation

#### 2.1.1 Plasma

Whole blood was collected in tubes with EDTA and immediately placed on ice. Within 15 min the blood was centrifuged (3000 g, 10 min at 4 °C) and plasma was collected and analyzed immediately or frozen (−80 °C) for storage until analysis.

#### 2.1.2 Cell culture

HEK293 cells were grown in a humidified incubator at 37 °C and 5% CO2 in DMEM (High Glucose, Gibco) with 5% fetal bovine serum (Hyclone) and 1% pen/strep. For experiments, 5000 cells were seeded in 100 μL media in a 96-well plate and incubated for 24 h before treatment. After treatment-incubation, 80 μL of supernatant was removed and analyzed immediately or frozen (−80 °C) for storage until analysis.

### 2.2 General reaction setup for ReactELISA

80 μL of sample was added to a microtiter plate (Greiner Bio-one Cellstar clear 96-well plate, 300 μL) containing 40 μL solution of 6 μM probe **3** in trichloroacetic acid solution (20 wt%). The microtiter plate was centrifuged at 3000 g for 10 min, and 100 μL of supernatant was transferred to another microtiter plate and sealed with a silicone seal (Thermo-Fischer Scientific). The plate was incubated at room temperature (rt) for 16–24 h. After the incubation time, 15 μL of carbonate buffer (1 M) was added to obtain a neutral pH. The resulting neutralized reaction samples can be used for either ELISA or AlphaLISA analysis.

### 2.3 ELISA analysis

100 μL of the neutralized reaction samples were transferred to a streptavidin-coated 96-well microtiter plate (Pierce™ Streptavidin Coated High Capacity) and incubated at rt for 1 h. After the incubation, the samples were removed followed by washing of the wells (3 × 300 μL 1 × PBS). 200 μL blocking buffer (2 w/v % skimmed milk protein (Premier Foods) in 1 × PBS (2% MPBS)) was added and allowed to incubate for 1 h at rt or overnight at 4 °C. This blocking buffer was removed followed by a washing step (3 × 300 μL 1 × PBS). 1.0 μg/mL product-specific antibody **MGAb** (in 2% MPBS) was then added and allowed to incubate for 2 h at rt. After a washing step (3 × 300 μL 1 × PBS), 100 μL polyclonal rabbit anti-mouse IgG-HRP diluted 1:4000 (Invitrogen) in 2% MPBS was added. The plate was incubated at rt for 1 h. Washing with 1 × PBS (3 × 300 μL) primed the plate for the addition of 100 μL TMB single solution (Life Technologies). The TMB solution was allowed to react for 5–15 min in complete darkness by covering with tinfoil. In order to quench the reaction, 50 μL 1 M H_2_SO_4_ was added, and the plate was analyzed within 10 min of the acid addition at OD_450_ subtracting OD_655_ (EnVision^®^ plate reader).

### 2.4 AlphaLISA analysis

5 μL of the neutralized reaction sample was transferred to a 384-well microtiter plate (PerkinElmer™ AlphaPlate-384) to this 25 μL of a mixture containing 12 μg/mL acceptor beads (PerkinElmer™ AlphaLISA anti-mouse IgG Acceptor beads), 24 μg/mL donor beads (PerkinElmer™ AlphaScreen Streptavidin Donor beads), and 0.12 μg/mL product-specific antibody **MGAb** was added. The plate was incubated in the dark for 5–18 h before being analyzed by reading emission at 615 nm after excitation at 680 nm (EnVision^®^ plate reader with Alpha module) [25].

### 2.5 UPLC-MS/MS analysis

200 μL plasma was treated with 100 μL TCA (20 wt%) and mixed by vortexing, followed by the addition of 200 μL H_2_O. The protein precipitated was removed by centrifugation and the supernatant filtered (UF-filter AcroPrep™ Advance 96 Filter plates 30K, Pall Corporation). To the filtrate, an aqueous solution of probe **3** was added to obtain the desired probe concentration. Samples were injected (10 μL, full loop injection overfill factor 4) onto an ACQUITY UPLC CSH Fluoro Phenyl Column (1.7 μm, 2.1 × 100 mm, Waters). Separation was achieved using a binary gradient at a flow rate of 0.4 mL/min. Mobile phase A was 0.2% formic acid in H_2_O, and mobile phase B was 0.1% formic acid in MeOH. Milli-Q H_2_O and LC-MS grade MeOH (Sigma-Aldrich) were used for eluents. Product **6** was separated using a linear gradient, starting at 10% mobile phase B for 0.5 min. The gradient was changed to 40% B over 0.1 min and then to 50% B over 0.9 min and kept there for 1.5 min. The gradient was then changed to 99% B over 0.1 min, kept there for 0.5 min before returning to the initial 10% B over 0.2 min. The retention time for **6** was 2.38 min. The mass spectrometer was set to operate in the positive ion mode with multiple reaction monitoring (MRM) experiments applied. **6** specific MRM transitions were 631.35/242.11 and 631.35/284.13 [m/z] for the quantification and qualification transition, respectively. All data acquisition and processing were performed using MassLynx 4.1 SCN 714 (Waters).

### 2.6 ^1^H NMR-based reactivity study

*o*-Phenylenediamine (oPD), Probe **1**, or Probe **3** was diluted in D_2_O to a concentration of 4 mM. From this solution, 0.5 mL was taken and transferred to an NMR tube. TCA was dissolved in D_2_O to a concentration of 6 wt% and 0.5 mL was added to the NMR tube and mixed by inversion. Aqueous solution of MG (100 mM, 21 μL, 2.1 μmol) was added to the NMR tube. Conversion to product was monitored by ^1^H NMR on a Bruker AVANCE III HD spectrometer running at 400 MHz and determined by comparison of integration of product and substrate peaks.

### 2.7 Antibody development

Product **6** specific antibodies from phage display were developed and obtained as previously described [24]. Product **6** specific antibody from immunization of mice was obtained by conjugating a biotin-free version of **6** (compound **18**, see Supporting Information) to the carrier protein KLH. Immunization was carried out in mice by New England Peptides, whom provided antibody-producing hybridoma cells. The antibody produced we termed **MGAb**. The hybridoma cells were grown in serum-free medium (Gibco Hybridoma SFM) supplemented with 0.5% pen/strep. After reaching a concentration of 5.4 × 10^5^ cells/mL with a viability ~97% the cells were split 1:2 and then incubated for 11 days. The cells were pelleted and the supernatant sterile filtered and purified on a 5 mL Protein G column (GE Healthcare) with a flow rate ~5 mL/min according to GE protocol. The antibody eluted with 0.1 M glycine buffer pH = 2.7 and was collected into 1 M TRIS-HCl pH = 9.0 (120 μL per mL eluate). Glycerol was added to a concentration of 15% and the antibody stored in aliquots at −80 °C.

### 2.8 UPLC-MS/MS-based MG quantification

The samples were extracted and derivatized with oPD according to the protocol of Rabbani and Thornalley [17]. Glassware was avoided; all reagents and sample extracts were stored in tubes and deep-well plates made of polypropylene. The chromatographic separation was performed by ultra-high-performance liquid chromatography using a Sciex Exion UHPLC system that consisted of binary pumps, a flow-through-needle sample manager set at 5 ± 2 °C and a column oven set at 45 ± 2 °C (AB Sciex, Foster City, CA). The mass spectrometer was a Sciex QTrap 6500+ triple-quadrupole instrument with a TurboIonSpray source operating in positive ion mode (ESI+). The separation was performed using an Acquity UPLC HSS C18 (1.8 μm, 2.1 mm I.D. × 100 mm) column (Waters, Milford, MA). A 10 μL volume of derivatized extract was injected onto the analytical column running 90% mobile phase A (0.1% FA in water) and 10% mobile phase B (0.1% FA in MeCN). The eluent was changed through a linear gradient to 40% B over 1 min, and then to 100% B over the next 1 min. Six min after injection, the gradient was returned to 10% B over 0.1 min, and the column was equilibrated for 1.9 min before the next injection, resulting in a total runtime of 8 min. The column flow rate was 400 μL/min, and the column temperature was maintained at 45 ± 2 °C. The optimal source parameters were: source temperature, 400 °C; ion spray voltage, 2.0 kV; nebulizer gas, 80 psi; heater gas, 80 psi; curtain gas, 20 psi. The optimal declustering potential was 80 V. The Q1 (*m/z*) > Q3 (*m/z*) transitions 145 > 128, 145 > 118, 145 > 92 and 145 > 77 of MG were monitored. The optimal collision energies were 32, 30, 31, 38 and 39 eV.

## 3 Results

### 3.1 Novel ReactELISA probes shows high reactivity with MG in human plasma

**Fig. 2:**
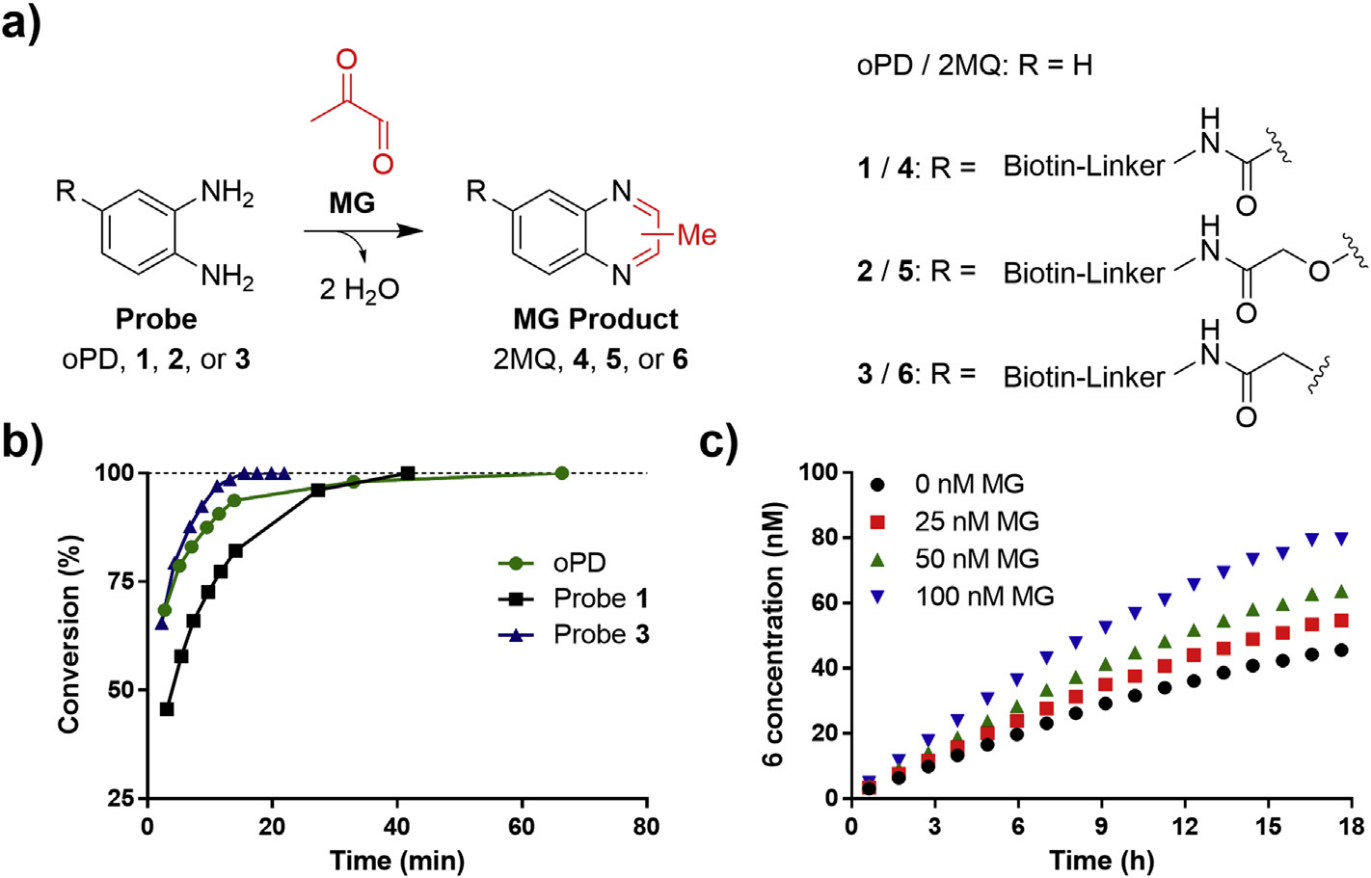
a) Chemical structure of oPD and ReactELISA probes **1**, **2**, and **3** and their reaction products with MG; 2MQ, **4**, **5**, and **6**, respectively. (Full chemical structure of probes in Fig. S1). b) Reactivity study of oPD, Probe **1** and **3**. oPD; Probe **1**, or Probe **3** (2 mM) was mixed with MG (2 mM) in D_2_O and conversion was determined from integration of probe peaks and product peaks in recorded ^1^H NMR spectra. c) Formation of product **6** in plasma spiked with 0–100 nM MG when using 1 μM probe **3**. Product concentration was determined using UPLC-MS/MS.

To develop the ReactELISA probe for chemoselective reaction with MG, we based the design on the known reaction between MG and oPD generating the stable product 2-methylquinoxaline (2MQ). Our expectation was this would be a suitable epitope for the antibody recognition (Fig. 2a).

The initially designed biotinylated probe **1** was synthesized using 3,4-diaminobenzoic acid with standard amide coupling reaction conditions (see Supporting Information). To evaluate the obtained probe **1**, the formation of product **4** upon addition of MG was monitored by ^1^H NMR spectroscopy and the rate of conversion compared to that of oPD (Fig. 2b). The ^1^H NMR experiment was carried out at supra-physiological concentrations (2 mM) and in a simpler matrix of water compared to a biological matrix as this was necessary to obtain a reliable signal. Under these conditions probe **1** showed good reactivity with MG but was inferior compared to oPD likely due to the electron withdrawing amide group conjugated to the oPD moiety of the probe.

To synthesize a more reactive probe, an electron-donating ether substituent was used to attach the biotin linker (Fig. 2a, compound **2**). Unfortunately, purification proved difficult as probe **2** decomposed rapidly upon exposure to air. This is consistent with another similarly structured derivatization agent for MG, 1,2-dimethoxy-4,5-diaminobenzene, which has shown instability and even oxidative degradations into MG possibly leading to false high MG readings [26]. As we wanted a robust assay with no concerns related to degradation, nor special precautions to avoid artificial MG contributions, no further attempts to obtain **2** or similar electron-rich probes were attempted.

As a compromise, we synthesized a probe with a methylene spacer between the amide functionality and the oPD moiety to avoid the electron-withdrawing effect as in probe **1** (Fig. 2a, probe **3**). This probe was easily synthesized, stable under standard conditions, and proved to be slightly superior to oPD in terms of reactivity towards MG (Fig. 2b).

Probe **3** was then evaluated under physiological conditions in human plasma. Using 1–100 μM probe **3** in plasma spiked with 0–100 nM MG good conversion from **3** to **6** as monitored by UPLC-MS/MS was achieved (Fig. 2c and Fig. S2). From the UPLC-MS/MS data, endogenous MG concentrations can be extrapolated using linear regression based on standard addition. The endogenous MG plasma used in Fig. 2c was determined to 126 nM (Fig. S2) which is within the expected plasma concentration [17].

### 3.2 Highly specific antibodies permits ReactELISA based quantification of MG without interference from other α-oxoaldehydes

Having developed a useful probe for measuring endogenous levels of MG we turned our attention to antibody development. A specific antibody for product **6** was initially obtained using phage-display techniques [24]. In short, product **6** was immobilized in streptavidin-coated wells, then a library of single-domain antibody displaying phages [27] were added to the wells allowing phage-antibodies to bind and nonbinding phages could be washed away. The binding phages were eluted and infected into *E. coli* which produced 313 colonies of which 12 produced phages with an affinity for product **6** (Fig. S8). The two best candidates, based on specificity towards product **6**, were selected for expression in *Leishmania terentolae* [28]*,* which yielded the single domain antibody as a dimer fused to a rabbit Fc domain. To evaluate the specificity of the obtained antibodies they were tested against product **6,** as well as product **7** obtained by reacting probe **3** with glyoxal (Fig. S9). One of the antibodies, termed **rFc 2** (Supporting Information), proved to have the most desirable specificity profile with selectivity towards products **6** over **7** and probe **3** (Fig. S9). However, the affinity of **rFc 2** seemed poor as a high antibody concentration was required and more than 25% of **3** needs to be converted into **6** in the assay to produce a significant signal (Fig. S10a).

**Fig. 3:**
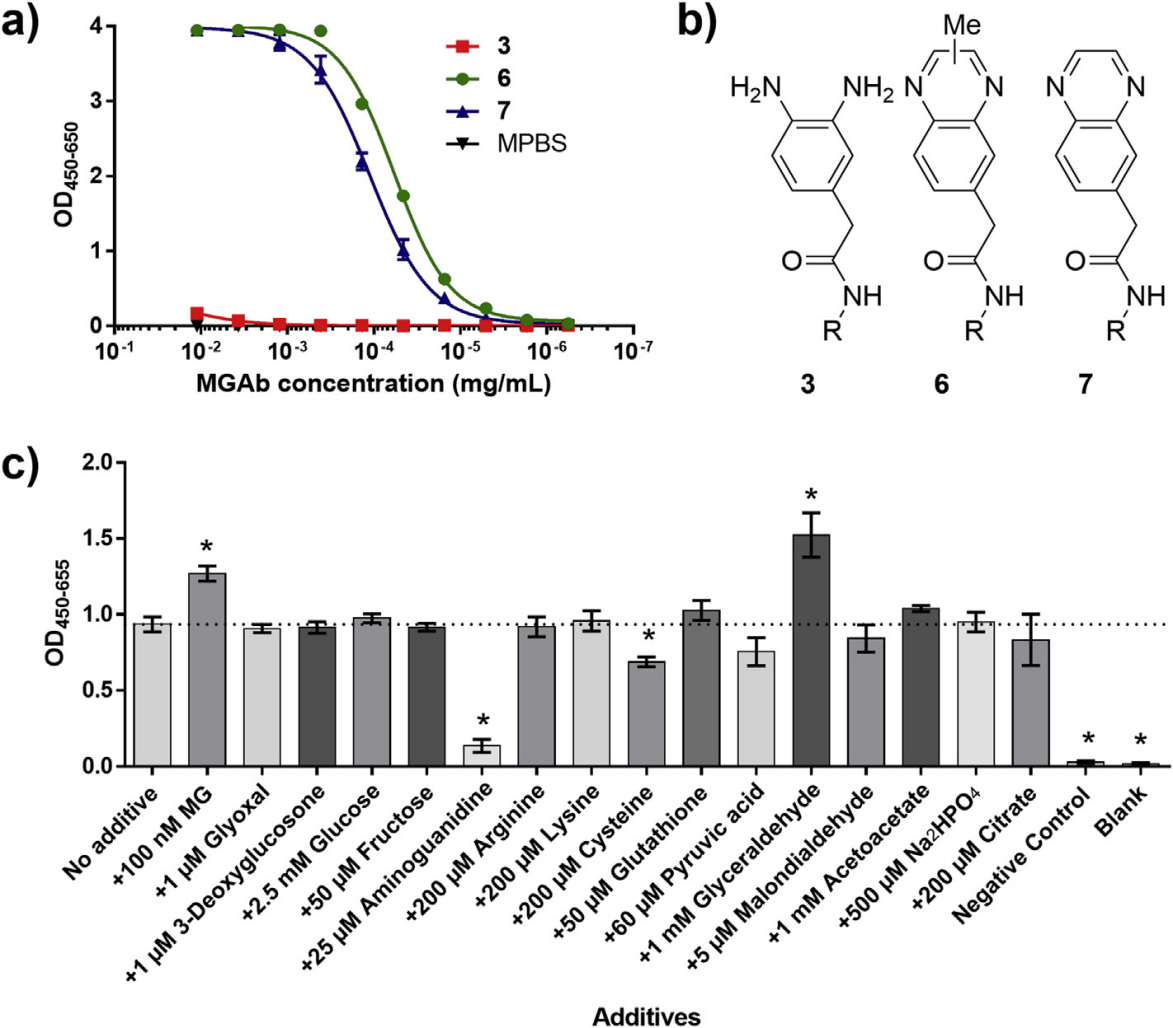
a) Specificity of murine antibody, **MGAb**. ELISA readout using varying concentrations of product **6** specific antibody **MGAb** as the primary antibody against wells coated with probe **3**, product **6**, glyoxal product **7**, and MPBS. b) Chemical structure of **3**, **6**, and **7**. c) Interference experiment. ELISA readout from 24 h reaction in PBS spiked with 400 nM MG and an additive in a biologically relevant concentration according to The Human Metabolome Database [30]. Negative control contains no MG. Blank contains neither probe nor MG. Reactions were made in triplicates. The error bars represent 1 standard deviation. * (p < 0.05 [*t*-test]) shows significance compared to no additive.

A stronger affinity antibody was obtained from murine immunization (Fig. S10). Mice were immunized with the carrier protein KLH conjugated to biotin-free product **6** (Compound **18**, Supporting Information). After standard monoclonal antibody production from the murine immunization we obtained a monoclonal mouse IgG which we termed **MGAb**. Due to the superiority of this antibody over the phage-display-obtained antibodies the applied antibody for following assays is henceforth **MGAb**. **MGAb** was specific for product **6** over probe **3**, but also binds to the glyoxal product **7** (Fig. 3a). This was, however, a minor concern as glyoxal products usually are formed in lower amounts than MG products and accordingly might not interfere with the assay [13].

To verify this, probe **3** was reacted with 400 nM MG in PBS together with glyoxal and 3-deoxyglycosone, both potentially interfering endogenous α-oxoaldehydes. Furthermore, a range of other endogenous and exogenouscompounds were tested for interference. The results showed that neither glyoxal nor 3-deoxyglycosone, had any significant impact on the results at the tested levels (Fig. 3c). However, cysteine showed to have some negative effect on the readout. This could be due to reversible binding of the thiol moiety of cysteine to MG which would effectively lower the reaction rate. It should be noted that the thiol containing GSH showed no effect, however, GSH was tested at lower concentrations compared to cysteine. As expected aminoguanidine, a well-known exogenous MG scavenger, almost completely abolished the signal. Glyceraldehyde showed an increased signal, probably due to its known degradation to MG [3]. It should be mentioned that glyceraldehyde was tested at 1 mM concentration in-line with earlier reported blood concentrations [29]. In hindsight, this concentration seemed unrealistically high and we accordingly developed a LC-MS/MS method for glyceraldehyde quantitation and determined that the true levels of glyceraldehyde in human plasma samples are orders of magnitude lower (13–20 nM) (method in Supporting Information). At these levels, glyceraldehyde is unlikely to cause significant interference with the assay.

### 3.3 Optimized ReactELISA conditions allow detection of low MG concentrations in human plasma

**Fig. 4:**
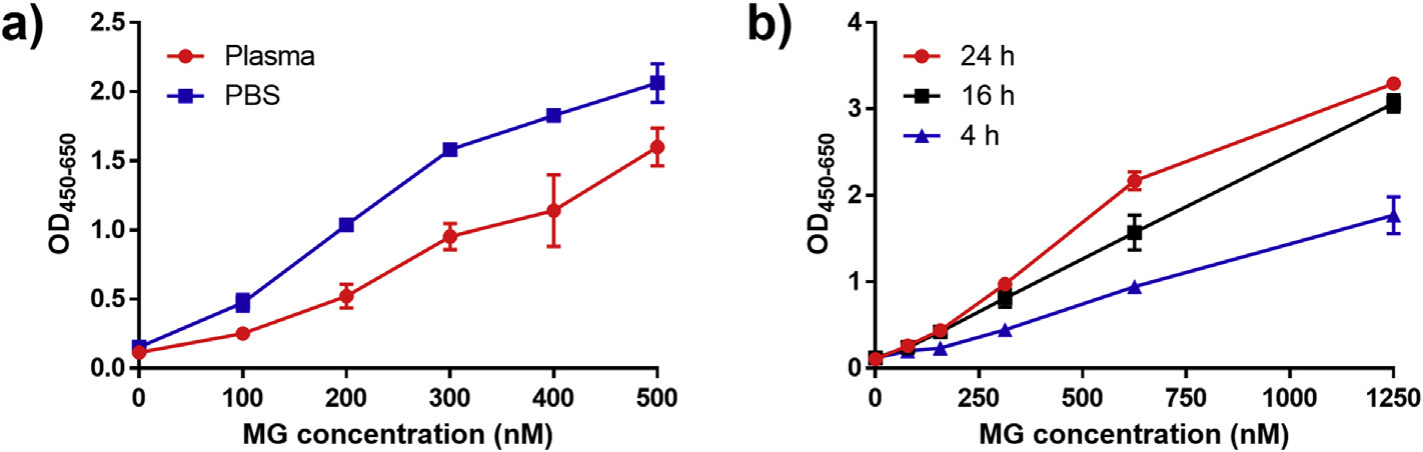
a) Reaction compared in PBS and plasma. ELISA readout after 24 h reaction with 1.25 μM probe **3** in PBS or plasma spiked with 0–500 nM MG. b) ELISA readout of reaction with 1.25 μM probe **3** in PBS spiked with 0–1250 nM MG for 4 h, 16 h, and 24 h. Reactions were made in duplicates. The error bars represent 1 standard deviation.

Having obtained a specific antibody for MG product **6**, we focused the attention toward the final setup. Initial experiments revealed that the efficiency of the assay was dependent on the complexity of the sample matrix as a higher readout was obtained in PBS compared to plasma (Fig. 4a). To run the reaction smoothly in blood plasma, proteins were accordingly removed by addition of trichloroacetic acid and centrifugation in the following experiments. It was also discovered that the reaction runs faster at low pH compared to neutral pH (Fig. S5). However, neutralization of the reaction mixture prior to immobilization in streptavidin wells lead to higher signal (Fig. S5), likely because the coated streptavidin might partially denature under the acidic conditions.

**Fig. 5:**
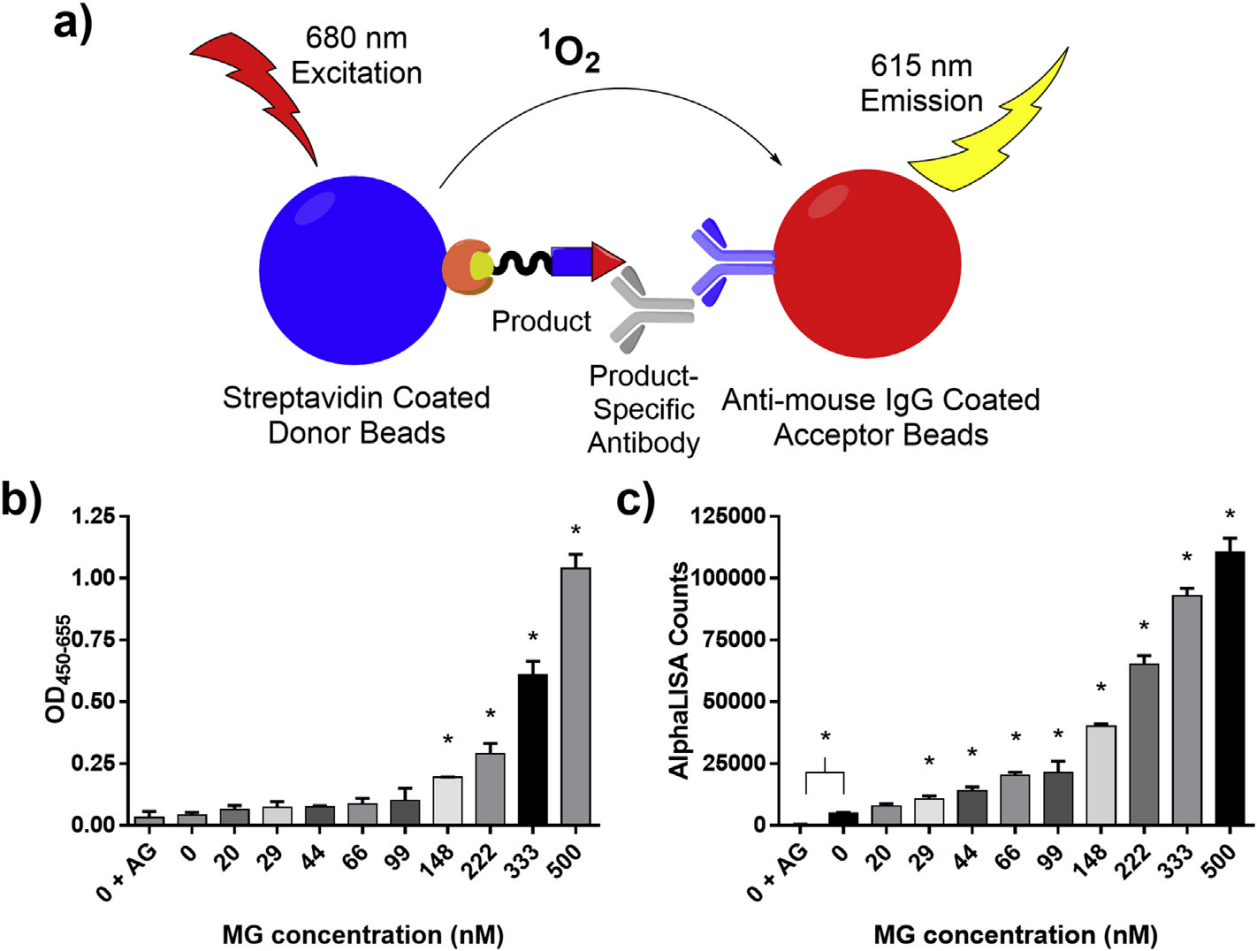
a) Schematic principle of AlphaLISA for MG. Binding of the anti-mouse IgG antibody-coated acceptor bead to the product-specific antibody and binding of the biotinylated product to the streptavidin covered donor beads bring the donor and acceptor beads into close proximity to each other upon binding of our antibody to the product. Once in close proximity, excitation of the donor beads at 680 nm release singlet oxygen which travels to the acceptor bead and result in emission at 615 nm. b) ELISA readout after 24 h reaction of 2 μM probe in plasma containing 47 nM endogenous MG spiked with 0–500 nM MG. c) AlphaLISA readout of the same samples as in b). Reactions were made in duplicates. The error bars represent 1 standard deviation. * (p < 0.05 [*t*-test]) shows significance compared to 0 nM MG spiked.

Reaction time and temperature were also investigated, and it was found that sufficient product could be obtained and detected after only 4 h of reaction in PBS with 300 nM MG (Fig. 4b). However, as product formation continued for at least 24 h, overnight incubation of 18–24 h was used in many of the following experiments for convenience (Fig. 4b). Longer incubation times and higher reaction temperature than rt have been reported to cause an overestimation of MG which might be a result of non-enzymatic degradation of monosaccharides and other cellular components over time and is thus avoided [17].

Finally, we examined the impact of probe concentration. If the probe concentration used in the reaction exceeds the binding capacity of the streptavidin in the wells, the excess (unreacted) probe in principle will outcompete the product for binding to the streptavidin and cause a reduced signal in the ReactELISA (Fig. S3). While a higher probe concentration will provide a faster and more complete reaction with MG, lower concentrations will lead to higher product to probe ratio which is what is assayed in the ReactELISA setup (Fig. S4). A probe concentration equal to the binding capacity of the streptavidin accordingly provides an optimal signal. In experiments, however, we use a small excess to ensure full saturation of the streptavidin.

Using optimized conditions 148 nM MG spiked into plasma gave significantly increased readout compared to plasma with no MG spiked and MG depleted plasma (Fig. 5b). MG was depleted from plasma by spiking with 5 mM aminoguanidine and incubation for 30 min. The endogenous MG concentration in the plasma used was measured using UPLC-MS/MS and determined to be 47 nM, which according to the literature is at the lower end of normal [17].

To finally validate the assay we determined the endogenous concentration of MG in four independent plasma samples by UPLC-MS/MS analysis. One sample was added increasing amounts of MG and from the ReactELISA data a calibration curve was constructed (Fig. S6). Using this curve the endogenous concentration of MG in the other three plasma samples were determined. Furthermore, the three independent plasma samples were spiked with MG at three levels (50–250 nM) and MG also determined by ReactELISA. The ratio between endogenous MG as determined by ReactELISA and UPLC-MS/MS varied between 71 and 120% (Fig. S6). The recovery varied between 62 and 126% at the three levels. This demonstrate that the method indeed can be used to measure endogenous and spiked MG in independent plasma samples. Larger clinical datasets are however needed to fully elucidate the potential of the assay.

### 3.4 ReactAlphaLISA for homogenous (no-wash), high sensitivity MG quantitation in biological samples

To increase the sensitivity of the ELISA as well as limit the number of steps in the analysis we converted the assay into a homogenous no-wash assay by combining the ReactELISA technology with the AlphaLISA principle. The combined ReactAlphaLISA principle for MG product measurements is illustrated in Fig. 5a.

Initially to obtain the optimal conditions for AlphaLISA for best signal, antibody and probe/product concentrations were titrated against each other (Fig. S7). Other parameters, such as donor and acceptor beads concentrations were chosen at the manufacturer's recommendations and not optimized further. Using the optimized concentrations, the assay was tested on the above-mentioned plasma samples, which had endogenous MG concentration of 47 nM. This plasma was further spiked with different levels of MG and compared to results from the standard ReactELISA protocol (Fig. 5b and c).

The ReactELISA using AlphaLISA (ReactAlphaLISA) for detection showed improved sensitivity compared to the standard ELISA detection protocol and was able to significantly distinguish between no MG spiked and 29 nM MG spiked in human plasma (Fig. 5c) while the standard ELISA needed around 150 nM (Fig. 5b). It also showed a significant difference between no MG spiked and MG depleted plasma (Fig. 5c), demonstrating that the assay can detect low endogenous plasma MG levels.

### 3.5 Buthionine sulfoximine treatment of HEK293 cells retards GLO1 activity which can be monitored by ReactAlphaLISA analysis of the culture media

**Fig. 6:**
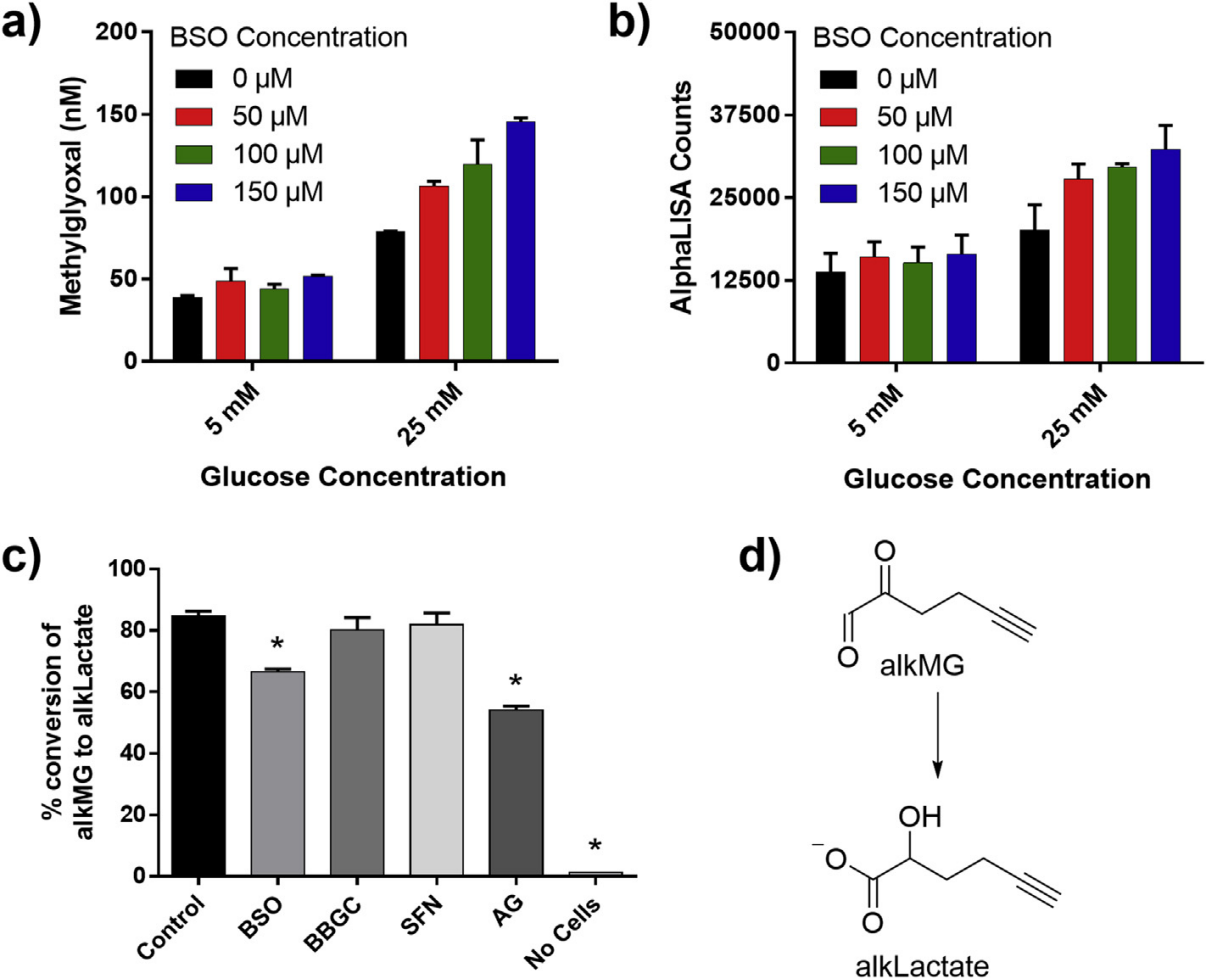
a) UPLC-MS/MS measurement of MG in cell culture media from HEK293 cells grown at low (5 mM) or high (25 mM) glucose concentration and treated with BSO (0–150 μM) for 42 h. Reactions were made in duplicates. b) ReactAlphaLISA readout of identical samples as in a). c) UPLC-MS/MS measured conversion of alkMG to alkLactate in HEK293 cells grown in DMEM. HEK293 cells were pretreated with vehicle (control), BSO (100 μM), BBGC (15 μM), SFN (5 μM), or AG (500 μM) for 24 h before addition of 500 μM alkMG and incubation for 4 h before work-up and analysis. Experiments were made in duplicates. d) Structure of alkMG and alkLactate. The error bars represent 1 standard deviation. * (p < 0.05 [*t*-test]) shows significance compared to control.

Being highly cell permeable, cellular levels of MG is reflected in the culture media. Accordingly, we moved on to investigate the ReactAlphaLISA assay in cell media as a simple phenotypic screen of cellular MG levels. HEK293 cells were grown in low and high glucose media while treated with 0–150 μM buthionine sulfoximine (BSO), an inhibitor of GSH synthesis, which has been shown to reduce GSH levels in HEK293 cells [31]. GSH reacts spontaneously with MG generating the hemithioacetal substrate for GLO1, thus reduced MG detoxification and increased MG levels were expected in response to BSO treatment. The results obtained from ReactAlphaLISA were compared to MG levels measured by the golden standard UPLC-MS/MS and showed a high correlation between the two methods (Fig. 6a and b) demonstrating that the ReactAlphaLISA assay can measure growth media MG efficiently as well as the data reflects the impact of BSO on MG levels. From the data it was observed that for HEK293 cells grown at low glucose concentration, no significant effect on MG level was seen after BSO treatment. In contrast, HEK293 cells grown at high glucose showed a dose-response relationship with increasing BSO concentration. The lack of any difference at low glucose might correlate to lower MG production still manageable by the glyoxalase/AKR systems, even at reduced GSH levels. At high glucose concentrations, an elevated MG level compared to low glucose is observed, even with no BSO treatment; a finding which is in agreement with the current literature [32].

To confirm that BSO indeed retards GLO1 catalyzed MG detoxification e.g. via a GSH hemithioacetal, we conducted an experiment with an in-house developed alkynylated MG probe (alkMG). AlkMG has previously been reported to mimic MG both with respect to electrophilic properties as well as it becomes detoxified into the corresponding alkynylated lactate (alkLactate) through the glyoxalase system (Fig. 6d) [33,34]. HEK293 cells where accordingly pretreated with BSO before incubation with 500 μM alkMG for 4 h and then the formation of alkLactate was quantified using UPLC-MS/MS as a measure of GLO1 activity. Vehicle treatment of the HEK293 cells resulted in 85% conversion of alkMG to alkLactate after 4 h, while pretreatment with BSO for 42 h significantly reduced the conversion to 67% (Fig. 6c), indicating that BSO does indeed reduce cellular GSH levels and accordingly GLO1 catalyzed MG detoxification. Curiously, pretreatment with S-*p*-bromobenzylglutathione (BBGC), a known GLO1 inhibitor [35], did not lead to a measurable decrease of alkLactate. Similarly, sulforaphane (SFN) a known GLO1 inducer [36] also showed no significant change from control experiments. The unexpected lack of response from BBGC might be due to the tested incubation time and concentration not being sufficient to perturb the system or the effect of the compound might be too insignificant for detection. The lack of an effect of SFN, on the other hand, could likely reflect that GLO1 already is highly expressed and additional expression will not change MG detoxification at the tested conditions. As expected, the direct MG scavenger aminoguanidine (AG) significantly reduced the amount of alkLactate produced.

## 4 Discussion

Collectively, these data clearly demonstrate that our novel ReactELISA is capable of measuring MG both in plasma as well as cell culture media as proxy for intracellular MG levels. We believe this is the first example of an operationally simple non-chromatography based assay to quantitate low nM MG levels in human biological samples. We anticipate that the assay can be adapted to other biological matrices as needed and hope this will open up for a broader usage of MG quantitations in biology and accordingly pave the way into a more detailed insight into those factors that regulate and control MG levels *in vivo* as well as the impact of MG on aging and diabetes related diseases. A further important aspect of the assay is its potential use as a homogenous and high-throughput amenable format for screening of compounds that may regulate MG metabolism in living cells. MG/GLO1 has been causally linked to several late diabetic complications and may be upstream most of the pathological mechanisms observed in diabetes [7,[37], [38], [39]]. Small molecules capable of modulating levels of this metabolite *in vivo* accordingly are highly relevant as probes to gain insight into the regulation and effect of MG *in vivo*. Furthermore, novel targets and compounds that regulate MG/GLO1 may have therapeutic potential. As example, the small molecule drug BSO has been used in several clinical trials to combat different forms of cancer and has recently been re-vitalized due to highly promising results when co-targeting thioredoxin systems in cancer and HIV treatment [[40], [41], [42]]. Discovery of further BSO analogues or other structures that regulate GSH and/or increase MG levels therefore are very relevant and should be obtainable with the assay. Furthermore, though BSO gave a robust response in the assay, even higher fold-change may be reachable by using cells devoid of one of the two major methylglyoxal detoxification systems e.g. a GLO1 knockout (GSH co-factor) or an AKR knockout (GSH independent) cell line in the assay [[43], [44], [45]]. Curiously, as both detoxification routes apparently are under the control of the DNA promoter antioxidant response element (ARE) activators or inhibitors of the KEAP1-NRF2 system should in principle be discoverable using any of the knockout cell lines [46,47]. NRF2 activators has attracted considerable attention as potential drugs for treating chronic diseases [48]. Interestingly, a phenotypic screen for NRF2 activators recently discovered a glycolysis inhibitor that initially increased cellular MG and then via a hormetic mechanism involving MG modification of KEAP1 activates NRF2 and ARE gene expression [49].

## 5 Conclusion

Historically reliable MG measurements in biological samples have only been obtainable via advanced chromatographic analysis, and still a high degree of uncertainty exists to the true levels of MG *in vivo*. We here present an operationally simple ReactELISA method that can be used in most biomolecular or medicinal laboratories, hopefully paving the way for a more widespread use of MG quantitation in biology. The setup is furthermore well suited for phenotypic screening of MG levels in cell culture for discovery of novel compounds capable of perturbing MG levels *in vivo*. Such compounds and their targets will be highly relevant to both gain insight into MG in biology, as well as they may function as potential leads for drugs against chronic and aging-related diseases.

## References

[1] Rabbani N., Thornalley P.J. (2012). Methylglyoxal, glyoxalase 1 and the dicarbonyl proteome. Amino Acids.

[2] Thornalley P.J., Langborg A., Minhas H.S. (1999). formation of glyoxal, methylglyoxal and 3-deoxyglucosone in the glycation of proteins by glucose. Biochem. J..

[3] Phillips S.A., Thornalley P.J. (1993). the formation of methylglyoxal from triose phosphates. Eur. J. Biochem..

[4] Ray S., Ray M. (1983). formation of methylglyoxal from aminoacetone by amine oxidase from goat plasma. J. Biol. Chem..

[5] Loidl-Stahlhofen A., Spitelier G. (1994). α-Hydroxyaldehydes, products of lipid peroxidation. Biochim. Biophys. Acta Lipids Lipid Metab..

[6] Kikuchi S., Shinpo K., Moriwaka F., Makita Z., Miyata T., Tashiro K. (1999). Neurotoxicity of methylglyoxal and 3-deoxyglucosone on cultured cortical neurons: synergism between glycation and oxidative stress, possibly involved in neurodegenerative diseases. J. Neurosci. Res..

[7] Bierhaus A., Fleming T., Stoyanov S., Leffler A., Babes A., Neacsu C., Sauer S.K., Eberhardt M., Schnölzer M., Lasitschka F. (2012). Methylglyoxal modification of Nav1.8 facilitates nociceptive neuron firing and causes hyperalgesia in diabetic neuropathy. Nat. Med..

[8] Chang T., Wu L. Methylglyoxal (2006). Oxidative stress, and hypertension. Can. J. Physiol. Pharmacol..

[9] Morcos M., Du X., Pfisterer F., Hutter H., Sayed A.A.R., Thornalley P., Ahmed N., Baynes J., Thorpe S., Kukudov G. (2008). Glyoxalase-1 prevents mitochondrial protein modification and enhances lifespan in Caenorhabditis elegans. Aging Cell.

[10] Brownlee M. (1995). Advanced protein glycosylation in diabetes and aging. Annu. Rev. Med..

[11] Ott C., Jacobs K., Haucke E., Navarrete Santos A., Grune T., Simm A. (2014). Role of advanced glycation end products in cellular signaling. Redox Biol..

[12] Xue M., Rabbani N., Thornalley P.J. (2011). Glyoxalase in ageing. Semin. Cell Dev. Biol..

[13] Rabbani N., Thornalley P.J. (2014). Dicarbonyl proteome and genome damage in metabolic and vascular disease. Biochem. Soc. Trans..

[14] Frandsen J.R., Narayanasamy P. (2018). Neuroprotection through flavonoid: enhancement of the glyoxalase pathway. Redox Biol..

[15] Vander Jagt D.L., Hunsaker L.A. (2003). Methylglyoxal metabolism and diabetic complications: roles of aldose reductase, glyoxalase-I, betaine aldehyde dehydrogenase and 2-oxoaldehyde dehydrogenase. Chem. Biol. Interact..

[16] Schumacher D., Morgenstern J., Oguchi Y., Volk N., Kopf S., Groener J.B., Nawroth P.P., Fleming T., Freichel M. (2018). Compensatory mechanisms for methylglyoxal detoxification in experimental & clinical diabetes. Mol. Metab..

[17] Rabbani N., Thornalley P.J. (2014). Measurement of methylglyoxal by stable isotopic dilution analysis LC-MS/MS with corroborative prediction in physiological samples. Nat. Protoc..

[18] Dhar A., Desai K., Liu J., Wu L. Methylglyoxal (2009). Protein binding and biological samples: are we getting the true measure?. J. Chromatogr. B Analyt. Technol. Biomed. Life Sci..

[19] Ramachandra Bhat L., Vedantham S., Krishnan U.M., Rayappan J.B.B. (2019). Methylglyoxal – an emerging biomarker for diabetes mellitus diagnosis and its detection methods. Biosens. Bioelectron..

[20] Scheijen J.L.J.M., Schalkwijk C.G. (2014). Quantification of glyoxal, methylglyoxal and 3-deoxyglucosone in blood and plasma by ultra performance liquid chromatography tandem mass spectrometry: evaluation of blood specimen. Clin. Chem. Lab. Med..

[21] Ma S., Subramanian R. (2006). Detecting and characterizing reactive metabolites by liquid chromatography/tandem mass spectrometry. J. Mass Spectrom..

[22] Wang S.-T., Lin Y., Spicer C.D., Stevens M.M. (2015). Bio-inspired maillard-like reactions enable a simple and sensitive assay for colorimetric detection of methylglyoxal. Chem. Commun..

[23] Wang T., Douglass E.F., Fitzgerald K.J., Spiegel D.A. (2013). A “Turn-On” fluorescent sensor for methylglyoxal. J. Am. Chem. Soc..

[24] Holmquist E.F., B Keiding U., Kold-Christensen R., Salomón T., Jørgensen K.A., Kristensen P., Poulsen T.B., Johannsen M. (2017). ReactELISA: monitoring a carbon nucleophilic metabolite by ELISA-a study of lipid metabolism. Anal. Chem..

[25] Bielefeld-Sevigny M. (2009). AlphaLISA immunoassay platform- the “no-Wash” high-throughput alternative to ELISA. Assay Drug Dev. Technol..

[26] McLellan A.C., Thornalley P.J. (1992). Synthesis and chromatography of 1,2-diamino-4,5-dimethoxybenzene, 6,7-dimethoxy-2-methylquinoxaline and 6,7-dimethoxy-2,3-dimethylquinoxaline for use in a liquid chromatographic fluorimetric assay of methylglyoxal. Anal. Chim. Acta.

[27] Mandrup O.A., Friis N.A., Lykkemark S., Just J., Kristensen P. (2013). A novel heavy domain antibody library with functionally optimized complementarity determining regions. PLoS One.

[28] Jørgensen M.L., Friis N.A., Just J., Madsen P., Petersen S.V., Kristensen P. (2014). Expression of single-chain variable fragments fused with the fc-region of rabbit IgG in Leishmania tarentolae. Microb. Cell Factories.

[29] Jonas A.J., Lin S.N., Conley S.B., Schneider J.A., Williams J.C., Caprioli R.C. (1989). Urine glyceraldehyde excretion is elevated in the renal fanconi syndrome. Kidney Int..

[30] Wishart D.S., Feunang Y.D., Marcu A., Guo A.C., Liang K., Vázquez-Fresno R., Sajed T., Johnson D., Li C., Karu N. (2018). HMDB 4.0: the human Metabolome Database for 2018. Nucleic Acids Res..

[31] Wang X., Jiang L., Ge L., Chen M., Yang G., Ji F., Zhong L., Guan Y., Liu X. (2015). Oxidative DNA damage induced by di-(2-ethylhexyl) phthalate in HEK-293 cell line. Environ. Toxicol. Pharmacol..

[32] Dhar A., Desai K., Kazachmov M., Yu P., Wu L. (2008). Methylglyoxal production in vascular smooth muscle cells from different metabolic precursors. Metabolism.

[33] Sibbersen C., Oxvig A.-M.S., Olesen S.B., Nielsen C.B., Galligan J.J., Jørgensen K.A., Palmfeldt J., Johannsen M. (2018). Profiling of methylglyoxal blood metabolism and advanced glycation end-product proteome using a chemical probe. ACS Chem. Biol..

[34] Salomón T., Sibbersen C., Hansen J., Britz D., Svart M.V., Voss T.S., Møller N., Gregersen N., Jørgensen K.A., Palmfeldt J. (2017). Ketone body acetoacetate buffers methylglyoxal via a non-enzymatic conversion during diabetic and dietary ketosis. Cell Chem. Biol..

[35] Thornalley P.J., Edwards L.G., Kang Y., Wyatt C., Davies N., Ladan M.J., Double J. (1996). Antitumour activity of S-P-bromobenzylglutathione cyclopentyl diester in vitro and in vivo. Inhibition of glyoxalase I and induction of apoptosis. Biochem. Pharmacol..

[36] Xue M., Rabbani N., Momiji H., Imbasi P., Anwar M.M., Kitteringham N., Park B.K., Souma T., Moriguchi T., Yamamoto M. (2012). Transcriptional control of glyoxalase 1 by Nrf2 provides a stress-responsive defence against dicarbonyl glycation. Biochem. J..

[37] Moraru A., Wiederstein J., Pfaff D., Fleming T., Miller A.K., Nawroth P., Teleman A.A. (2018). Elevated levels of the reactive metabolite methylglyoxal recapitulate progression of type 2 diabetes. Cell Metabol..

[38] Giacco F., Du X., D'Agati V.D., Milne R., Sui G., Geoffrion M., Brownlee M. (2014). Knockdown of glyoxalase 1 mimics diabetic nephropathy in nondiabetic mice. Diabetes.

[39] Brouwers O., Niessen P.M.G., Miyata T., Østergaard J.A., Flyvbjerg A., Peutz-Kootstra C.J., Sieber J., Mundel P.H., Brownlee M., Janssen B.J.A. (2014). Glyoxalase-1 overexpression reduces endothelial dysfunction and attenuates early renal impairment in a rat model of diabetes. Diabetologia.

[40] Cramer S.L., Saha A., Liu J., Tadi S., Tiziani S., Yan W., Triplett K., Lamb C., Alters S.E., Rowlinson S. (2017). Systemic Depletion of L-Cyst(e)ine with Cyst(e)inase Increases Reactive Oxygen Species and Suppresses Tumor Growth. Nat. Med..

[41] Harris I.S., Treloar A.E., Inoue S., Sasaki M., Gorrini C., Lee K.C., Yung K.Y., Brenner D., Knobbe-Thomsen C.B., Cox M.A. (2015). Glutathione and thioredoxin antioxidant pathways synergize to drive cancer initiation and progression. Cancer Cell.

[42] Benhar M., Shytaj I.L., Stamler J.S., Savarino A. (2016). Dual targeting of the thioredoxin and glutathione systems in cancer and HIV. J. Clin. Investig..

[43] Niimi N., Yako H., Takaku S., Kato H., Matsumoto T., Nishito Y., Watabe K., Ogasawara S., Mizukami H., Yagihashi S. (2018). A spontaneously immortalized schwann cell line from aldose reductase-deficient mice as a useful tool for studying polyol pathway and aldehyde metabolism. J. Neurochem..

[44] Morgenstern J., Fleming T., Schumacher D., Eckstein V., Freichel M., Herzig S., Nawroth P. (2017). Loss of glyoxalase 1 induces compensatory mechanism to achieve dicarbonyl detoxification in mammalian schwann cells. J. Biol. Chem..

[45] Baba S.P., Barski O.A., Ahmed Y., O'Toole T.E., Conklin D.J., Bhatnagar A., Srivastava S. (2009). Reductive metabolism of age precursors: a metabolic route for preventing age accumulation in cardiovascular tissue. Diabetes.

[46] Raghunath A., Sundarraj K., Nagarajan R., Arfuso F., Bian J., Kumar A.P., Sethi G., Perumal E. (2018). Antioxidant response elements: discovery, classes, regulation and potential applications. Redox Biol..

[47] Xue M., Rabbani N., Momiji H., Imbasi P., Anwar M.M., Kitteringham N., Park B.K., Souma T., Moriguchi T., Yamamoto M. (2012). Transcriptional control of glyoxalase 1 by Nrf2 provides a stress-responsive defence against dicarbonyl glycation. Biochem. J..

[48] Cuadrado A., Rojo A.I., Wells G., Hayes J.D., Cousin S.P., Rumsey W.L., Attucks O.C., Franklin S., Levonen A.-L., Kensler T.W. (2019). Therapeutic targeting of the NRF2 and KEAP1 partnership in chronic diseases. Nat. Rev. Drug Discov..

[49] Bollong M.J., Lee G., Coukos J.S., Yun H., Zambaldo C., Chang J.W., Chin E.N., Ahmad I., Chatterjee A.K., Lairson L.L. (2018). A metabolite-derived protein modification integrates glycolysis with KEAP1–NRF2 signalling. Nature.

