# Supplementary material for: ReactELISA method for quantifying methylglyoxal levels in plasma and cell cultures
Source: Redox Biol. 2019 Jun 11;26:101252. doi: 10.1016/j.redox.2019.101252 (PMC6604041; doi:10.1016/j.redox.2019.101252)
Supplement: Multimedia component 1 [file mmc1.pdf]

Supporting Information

ReactELISA Method for Quantifying Methylglyoxal Levels in Plasma and Cell Cultures

Rasmus Kold-Christensen,[a,b] Karina Kragh Jensen,[a] Emil Smedegård-Holmquist,[a,b] Lambert Kristiansen Sørensen,[a] Jakob Hansen,[a] Karl Anker Jørgensen,[b] Peter Kristensen,[c,†] and Mogens Johannsen[a*]

[a] Department of Forensic Medicine, Aarhus University, Palle Juul-Jensens Boulevard 99, 8200 Aarhus N, Denmark

[b] Department of Chemistry, Aarhus University, Langelandsgade 140, 8000 Aarhus C, Denmark

[c] Department of Engineering, Aarhus University, Gustav Wieds Vej 10, 8000 Aarhus C, Denmark

† Present address: Department of Chemistry and Bioscience Aalborg University, Frederik Bajers Vej 7, 9220 Aalborg, Denmark

Table of Contents

Supporting Figures 2
General Protocol for ReactELISA for MG 9
Quantitative Determination of Glyceraldehyde 11
AlkLactate Quantification 13
Synthesis 14
NMR 21
References 26

Supporting Figures

1 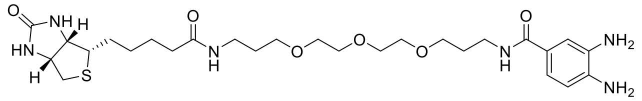

2 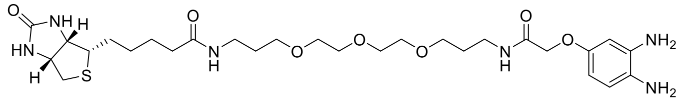

3 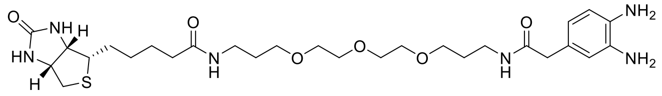

6 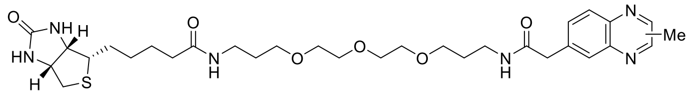

7 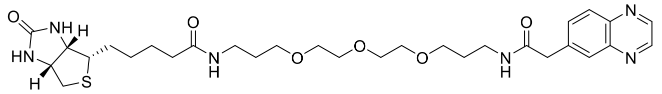

Figure S1. Chemical Structure of Probes and Products. Chemical structure of probes **1**, **2** (not synthesized), and **3**, as well as the MG product **6** and the glyoxal product **7**.

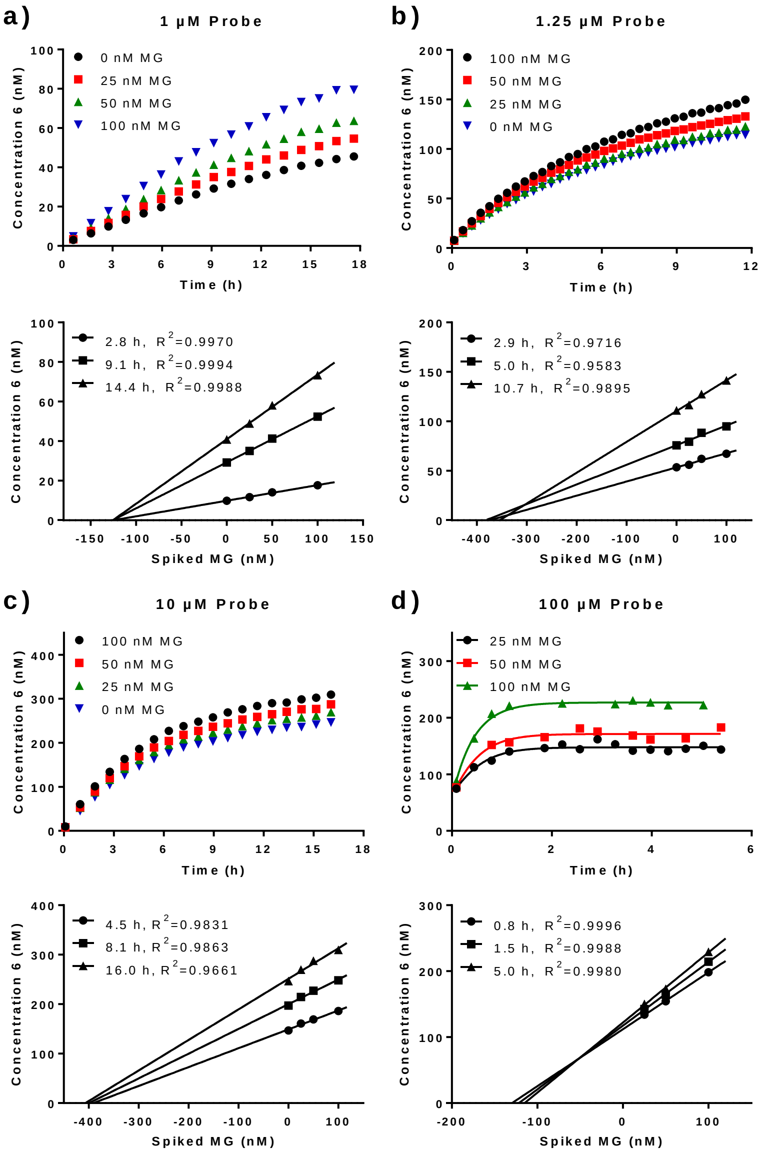

Figure S2. UPLC-MS/MS Data for Reaction in Four Different Plasma Samples. Top graphs: Formation of product **6** in different plasma samples spiked with 0-100 nM MG when using varying concentrations of probe **3**. Bottom graphs: Concentration of **6** from top graphs plotted against spiked MG concentration and fitted with linear regression to extrapolate endogenous plasma MG concentrations at the intercept between the linear regression and the x-axis. a) 1 µM of probe **3**, endogenous MG plasma concentration of 126 nM. b) 1.25 µM of probe **3**, endogenous MG plasma concentration of 371 nM. c) 10 µM of probe **3**, endogenous MG plasma concentration of 399 nM. d). 100 µM of probe **3**, endogenous MG plasma concentration of 122 nM. Concentration of **6** was determined using UPLC-MS/MS.

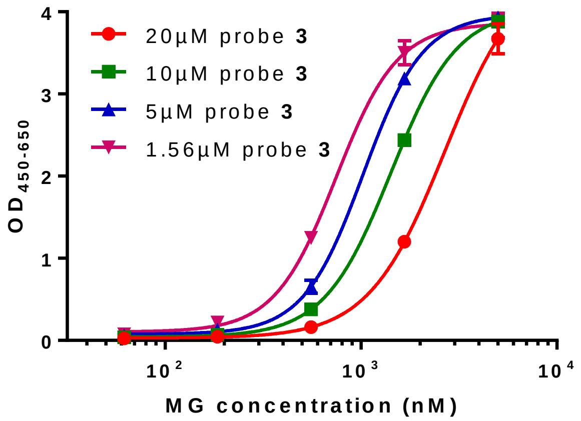

Figure S3. Probe Concentration Screening. Reaction with 1.56-20 µM probe **3** and 62-5000 nM MG in PBS followed by standard ELISA detection. The streptavidin plates (Pierce™ Streptavidin Coated High Capacity 96-well plates) had a binding capacity of ~125 pmol/well according to manufacturer. With a coating volume of 100 µL 1.56, 5, 10, 20 µM probe is a binding excess of 1.25, 4, 8, 16, respectively.

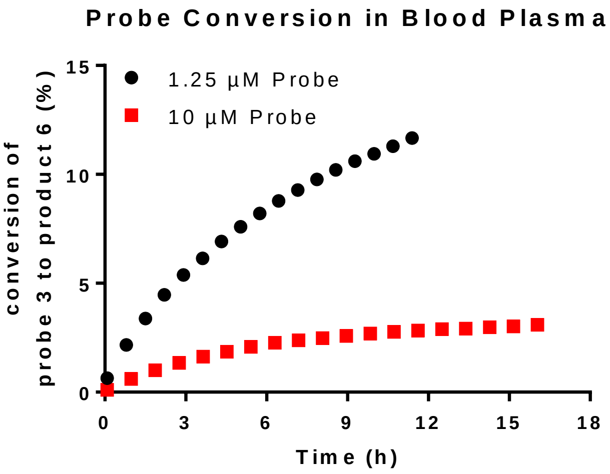

Figure S4. Product/Probe Ratio. Calculated percentage conversion of probe **3** to product **6** at a given time based on UPLC-MS/MS results from Figure S2b and S2c. While higher probe **3** concentration leads to higher quantity of product **6** formation in a given time (Figure S2), lower probe **3** concentrations lead to higher product **6** to probe **3** ratio (this figure) which is what is measured in ReactELISA. Figure S2b and S2c were selected for these calculations as they had similar endogenous plasma concentrations.

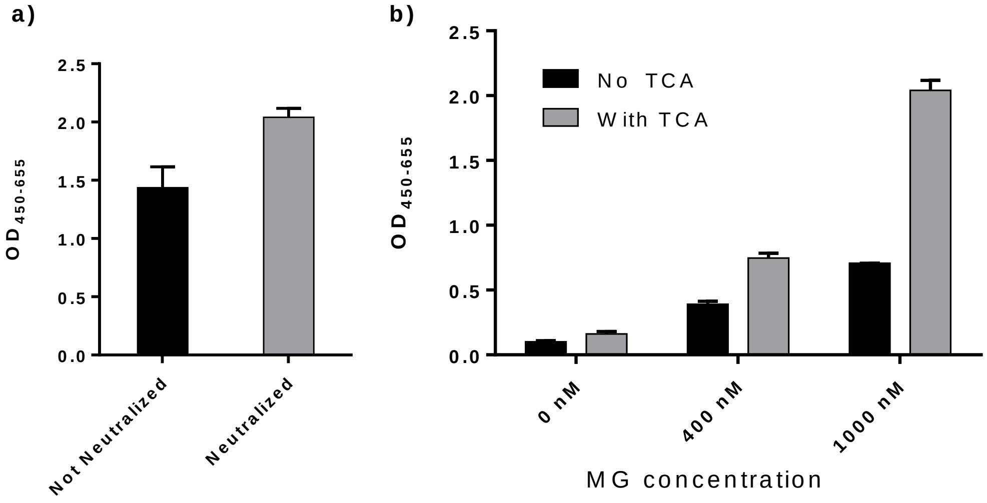

Figure S5. TCA and Neutralization. a) Reaction with 1.25 µM probe **3** and 1000 nM MG in PBS with 4 wt.% TCA followed by neutralization with carbonate buffer or no neutralization before coating in streptavidin wells following standard ELISA protocol. b) Reaction between 1.25 µM probe **3** and 0, 400, or 1000 nM MG spiked in PBS with and without addition of TCA to a final TCA concentration of 4 wt.% followed by neutralization. Reactions were made in duplicates. The error bars represent one standard deviation.

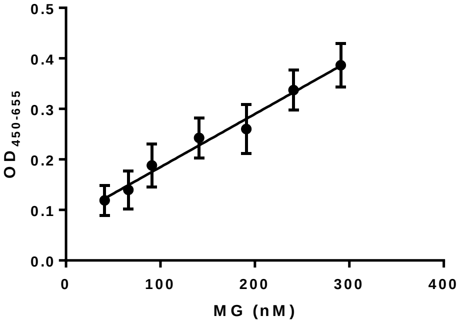

Spiked MG (nM)	$OD_{450-655}$	MG (nM) (ELISA)[d]	MG (nM) (UPLC-MS/MS)[e]	Ratio %[f]	Recovery
0[a]	0.11±0.01	40±11	39	102±28	-
50[a]	0.15±0.01	70±5	-	-	62±10
150[a]	0.22±0.02	149±15	-	-	73±10
250[a]	0.34±0.03	253±29	-	-	85±12
0[b]	0.14±0.02	52±21	43	120±49	-
50[b]	0.19±0.00	106±3	-	-	126±7
150[b]	0.23±0.04	161±32	-	-	79±21
250[b]	0.39±0.06	282±59	-	-	95±24
0[c]	0.11±0.01	34±11	48	71±23	-
50[c]	0.16±0.02	84±10	-	-	73±19
150[c]	0.28±0.02	193±20	-	-	96±13
250[c]	0.42±0.01	329±9	-	-	111±3

Figure S6. Validation and Recovery. Calibration curve made from spiking plasma with 0-250 nM MG. The endogenous plasma concentration was determined by UPLC-MS/MS to 41 nM and the curve adjusted accordingly. Table: [a], [b], and [c] are independent plasma samples. [d] MG concentration determined by ELISA using the calibration curve. [e] Endogenous levels of MG in plasma samples [a], [b], and [c] were determined by UPLC-MS/MS. [f] Concentration ratio of MG from [d] compared to [e]. All reactions were made in triplicates. The error bars represent one standard deviation.

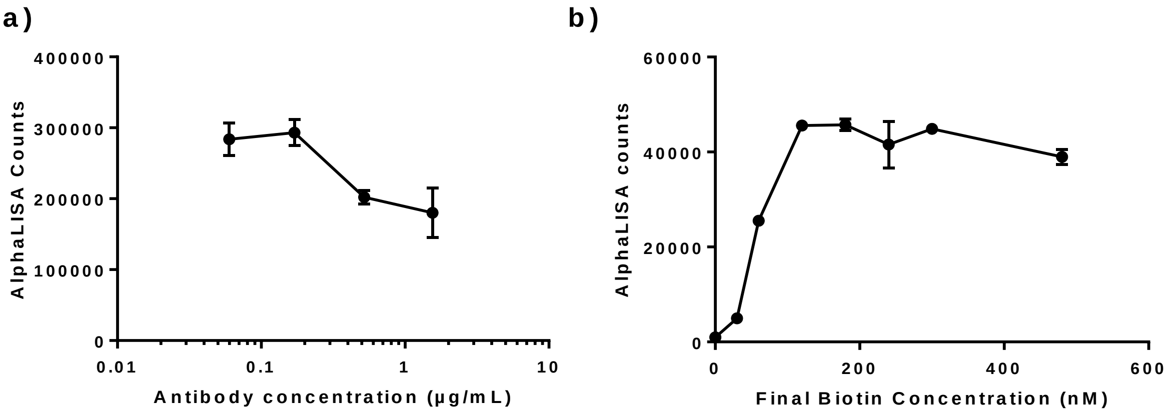

Figure S7. Optimization for AlphaLISA Conditions. a) AlphaLISA results using varying concentrations of antibody with a constant amount (60 nM final assay concentration) of product **6**. b) AlphaLISA results using varying concentrations of a 10% product **6**/probe **3** mix with a constant concentration of antibody (0.1 µg/mL). Experiment was made in duplicates. The error bars represent one standard deviation.

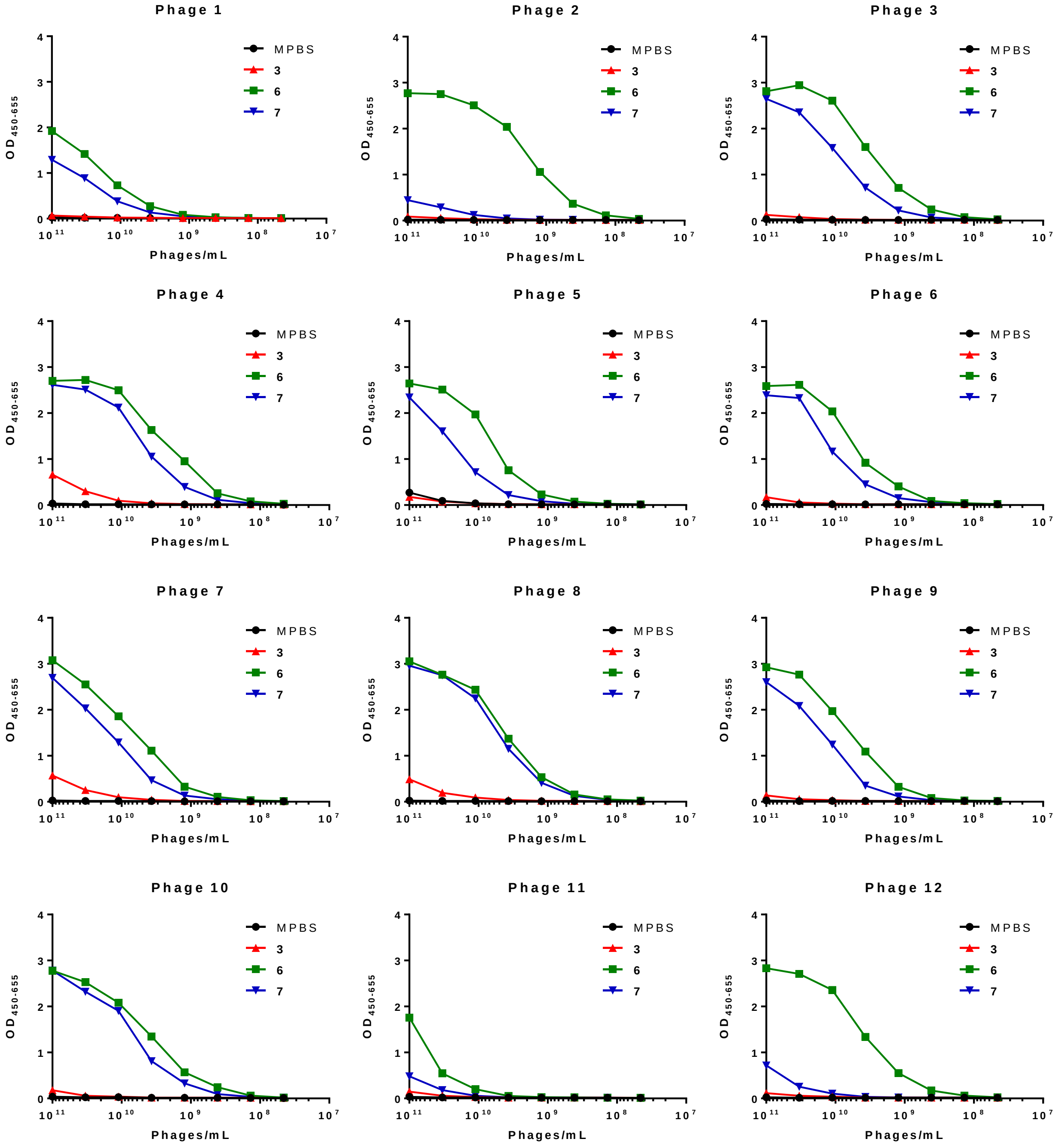

Figure S8. Specificity of Phage Antibodies. ELISA against **3**, **6**, **7**, and MPBS (control) using obtained phage antibodies (**Phage 1-12**) from phage display as the primary antibody and anti-m13-HRP (GE Healthcare) as the secondary antibody. **Phage 2** and **Phage 12** were selected for expression in *Leishmania terentolae* due to their superior specificity towards product **6**.

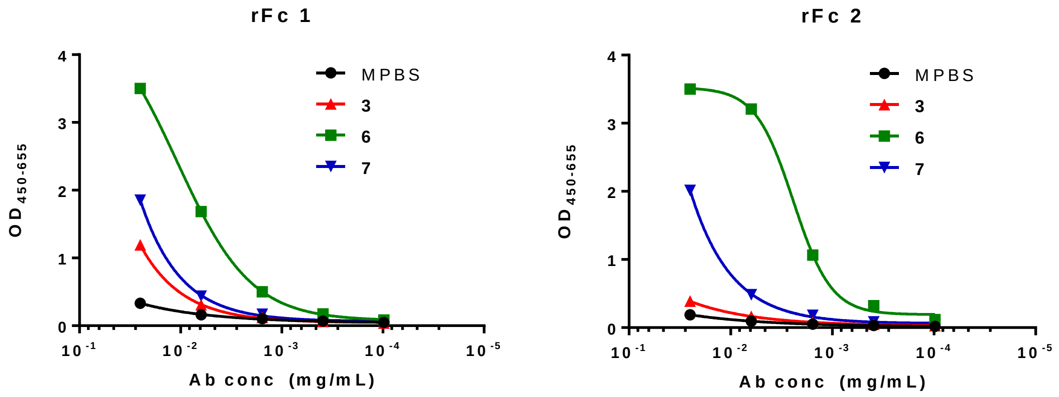

Figure S9. Specificity of rFc Antibodies. ELISA against **3**, **6**, **7**, and MPBS (control) using obtained antibodies after expression in *Leishmania terentolae* of **Phage 2** and **Phage 12** to obtained **rFc 1** and **rFc 2**, respectively.

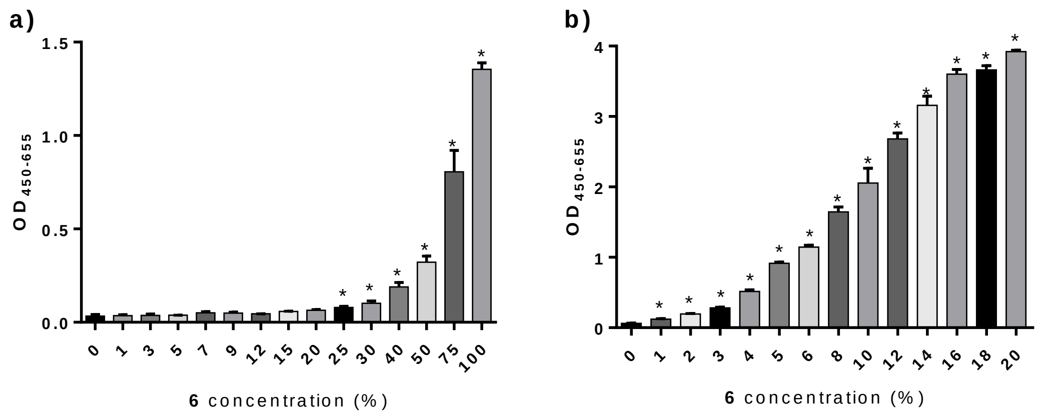

Figure S10. Sensitivity of antibodies against 6. ELISA readout with a coating of a mixture of **6** and **3** containing 0-100% **6**. For the primary antibody were used a) **rFc 2** or b) **MGAb** obtained from immunization. Reactions were made in duplicates for a) and triplicates for b). The error bars represent one standard deviation. . * (p <0.05 [t-test]) shows significance compared to 0% **6** concentration.

General Protocol for ReactELISA for MG

The ReactELISA reaction was performed in Greiner Bio-one Cellstar clear 96-well plates, 300 µL. The reaction was scalable down to 20 µL sample if desired as AlphaLISA only requires 5 µL, however 100 µL is needed for ELISA detection.

1) 50 µL of a solution with 6 µM Probe in TCA (20wt.%) was added to the plate.
2) 100 µL sample (plasma, cell culture media, PBS) was added to the plate.
   a. For samples with high protein content/precipitate such as plasma, mixing with microtiter plate shaker or in some cases by pipetting was necessary.
3) Incubation for 5 min.
4) Centrifugation at 3000 g for 10 min at rt.
5) 100 µL supernatant was moved to a new 96-well plate.
6) The plate was sealed using silicon sealing mat and incubated 16-24 h at rt.
7) 15 µL 1 M carbonate solution was added to obtain a neutral pH.
   a. Universal pH indicator paper showing pH = 6-8.
8) The samples were analyzed by ELISA or AlphaLISA immediately following neutralization.

ELISA detection

ELISA was done in Pierce™ Streptavidin Coated High Capacity 96-well plates and the OD was measured on a EnVision plate reader. 2% MPBS was obtained by adding 1 g skim milk powder (Sigma-Aldrich) in 50 mL PBS followed by vortexing. Secondary antibody was polyclonal rabbit anti-mouse IgG-HRP (Invitrogen). The TMB solution was TMB Single Solution (Ready-to-Use) (Invitrogen).

1) 100 µL of the neutralized solution was moved to a streptavidin coated well in a 96-well plate and sealed using sealing tape.
2) Incubation for 1 h at rt to allow coating of probe/product mixture.
3) The solution was removed from the plate and the plate was washed with 3 x 300 µL PBS.
4) The plate was blocked by adding 300 µL 2% MPBS and incubated for 1 h at rt or overnight at 4 °C.
5) The blocking solution was removed and the plate washed with 3 x 300 µL PBS.
6) 100 µL 1.0 µg/mL product specific antibody in 2% MPBS was added and the plate was incubated for 2 h at rt.
7) The primary antibody solution was removed and the plate was washed with 3 x 300 µL PBS.
8) 100 µL secondary antibody diluted 1:4000 in 2% MPBS was added and the plate was incubated for 1 h at rt.
9) The secondary antibody solution was removed and the plate was washed with 3 x 300 µL PBS.
10) 100 µL TMB solution was added and the TMB was allowed to react in darkness by covering the plate with tinfoil.
11) TMB reacted for 5-15 min.
12) The reaction was stopped by adding 50 µL 1 M H_2SO_4.
13) The plate was read within 10 min of stopping the TMB reaction at OD_{450} and subtracted by OD_{655}.

AlphaLISA detection

AlphaLISA was performed in PerkinElmer™ AlphaPlate-384 384-well plates. Readout was measured on an EnVision plate reader with Alpha module. Acceptor beads were AlphaLISA anti-mouse IgG Acceptor beads (PerkinElmer). Donor beads were AlphaScreen Streptavidin Donor beads (PerkinElmer). The buffer for alphaLISA was Immunoassay buffer (PerkinElmer).

1) 5 µL of the neutralized reaction sample were transferred to a well in a 384-well microtiter plate.
2) To this was added 25 µL of a solution containing 12 µg/mL acceptor beads, 24 µg/mL donor beads, and 0.12 µg/mL product specific antibody in buffer.
    a. This addition and solution was handled under reduced lighting due to light sensitivity of the donor beads.[i]
3) The plate was incubated in darkness for 5-18 h before being analyzed by reading emission at 615 nm after excitation at 680 nm.
    a. Readout continued to increase with incubation time for at least 24 h. However, after 5 h signal to background did not improve and after 18 h signal to background ratio fell.

Quantitative determination of glyceraldehyde in plasma by liquid chromatography tandem mass spectrometry (LC-MS/MS)

Standards and reagents
Glyceraldehyde-$^{13}C_3$ was obtained from Toronto Research Chemicals Inc. (TRC) (North York, Canada). Glyceraldehyde, 2,4-dinitrophenylhydrazine (DNPH), sodium azide (NaN_3), diethylenetriaminepentaacetic acid (DETAPAC) and trichloroacetic acid (TCA) were purchased from Sigma-Aldrich (Schnelldorf, Germany). Sodium chloride (NaCl), acetonitrile (MeCN), formic acid (FA) and hydrochloric acid (HCl) were obtained from Merck (Darmstadt, Germany). Water was purified using a Direct-Q 3 apparatus (Millipore, Bedford, MA). Stock solutions of glyceraldehyde and glyceraldehyde-$^{13}C_3$ (100 µM) were prepared in water. Standard solutions containing 0.01, 0.1, 1, 2.5, 5, 7.5 and 10 µM glyceraldehyde and a 0.5 µM solution of glyceraldehyde-$^{13}C_3$ (stable isotope labelled internal standard (SIL-IS) solution) were prepared by diluting the stock solutions with water. A 3% NaN_3 solution and a TCA solution containing 20% (w/v) TCA and 0.9% (w/v) NaCl was prepared in water. A 0.5 mM DETAPAC solution was prepared in 0.2 M HCl. A 15 mM DNPH stock solution was prepared in MeCN. A 2.3 mM derivatization solution was prepared by mixing 275 µL of DNPH stock solution, 300 µL of NaN_3 solution and 1200 µL of DETAPAC solution.

Instrumentation and materials
The ultra-high performance liquid chromatography system was a Sciex Exion UHPLC system that consisted of binary pumps, a flow-through-needle sample manager set at 5 ± 2°C and a column oven set at 45 ± 2°C (AB Sciex, Foster City, CA). The mass spectrometer was a Sciex QTrap 6500+ triple-quadrupole instrument with a TurboIonSpray source. The separation was performed using an Acquity UPLC HSS C18 (1.8 µm, 2.1 mm I.D. × 100 mm) column (Waters, Milford, MA). Sample treatments were performed in 2 mL Safe-Lock tubes and 1 mL 96-well plates from Eppendorf (Hamburg, Germany). All reagents were stored in polypropylene tubes with screw cap.

Sample preparation
A 80 µL volume of plasma was transferred to a 2 mL Safe-Lock tube and mixed with 40 µL of the 20% TCA solution. The suspension was mixed with 40 µL of water, 40 µL of SIL-IS solution and then centrifuged at 13,000 g for 5 min. A 70 µL volume of the supernatant was transferred to a deep well plate and mixed with 30 µL of the derivatization solution. After 4 h in the dark at ambient temperature the sample solution was mixed with 100 µL of MeCN.

Liquid chromatography tandem mass spectrometry (LC-MS/MS)
A 10-µL volume of derivatized extract was injected onto the analytical column running 50% mobile phase A (0.1% FA in water) and 50% mobile phase B (0.1% FA in MeCN). The eluent was changed through a linear gradient to 100% B over 2 min. Six minutes after injection, the gradient was returned to 50% B over 0.1 min, and the column was equilibrated for 1.9 min before the next injection, resulting in a total runtime of 8 min. The column flow rate was 400 µL/min, and the column temperature was maintained at 45 ± 2 °C. The mass spectrometer was operated in negative ion mode (ESI-) at unit mass resolution. The optimal source parameters were: source temperature, 600 °C; ionspray voltage, -3.0 kV; nebulizer gas (gas-1), 60 psi; heater gas (gas-2), 60 psi; curtain gas, 20 psi. The optimal declustering potential was -80 V. Selected reaction monitoring (SRM) was applied using the Q1 (*m/z*) > Q3 (*m/z*) transitions 269 > 105 and 269 > 239 for glyceraldehyde, and 272 > 108 and 272 > 241 for glyceraldehyde-$^{13}C_3$. The optimal collision energy was 15 eV in all cases. Nitrogen was used for the collision-induced dissociation. The dwell time was 25 msec and at least 12 data points were obtained across the peaks. Data acquisition and processing were performed using Analyst 1.7 (AB Sciex).

Calibration

Pure aqueous calibrants were used for the construction of 7-point calibration curves. The calibrants were treated according to the above procedure, except that 80 µL of plasma was replaced by 80 µL of standard solutions. The calibration curves were created by weighted (1/x) regression analysis of the SIL-IS normalised peak areas (analyte area/IS area).

AlkLactate Quantification

AlkLactate was quantified in spent growth media obtained from the culturing of HEK293 cells in DMEM (High Glucose, Gibco) supplemented with 5% fetal bovine serum (Hyclone) and 1% pen/strep. In a 96-well microtiter plate, 10000 cells were seeded and incubated for 24 h before treatment with buthionine sulfoximine (BSO) (100 µM), S-*p*-bromobenzylglutathione (BBGC) (15 µM), sulforaphane (SFN) (5 µM), AG (500 µM), or no treatment (control). After a further 24 h incubation, cells were treated with alkMG (500 µM) for 4 h.

For sample preparation, 40 µL cell media sample was added to 40 µL MeOH and 80 µL acetonitrile followed by vortex mixing. After 2 min of incubation and centrifugation for 5 min (12000 *g*) at rt, a 40 µL aliquot of the supernatant was diluted with 960 µL water with 0.3% formic acid to yield the final sample for UPLC-MS/MS. For calibration curves, a separate set of calibrant samples was prepared by spiking unused cell media with known amounts of alkLactate reference compound (at levels [µM]: 200, 50, 12.5, 3.13, 0.78, and 0).

Samples were injected (20 µL) into a UPLC system (Waters Acquity) and separated on a UPLC HSS-T3 column (Waters Acquity UPLC HSS T3 1.8µm, 2.1 x 100mm). An 11 min gradient LC-profile was used starting with 100% solvent A (water + 0.1% formic acid), linear gradient to 50% solvent A and 50% solvent B (acetonitrile + 0.1% formic acid) over 3.5 min, changed to 95% solvent B at 3.6 min and maintained until 5.1 min, followed by 100% solvent A from 5.1 to 11 min.

AlkLactate was detected using a triple quadrupole mass spectrometer instrument (Waters Aquity Xevo TQ-S) operated in the MRM mode using electrospray ionization in the negative mode. General MS settings: capillary voltage 2.0 kV; source temperature 150 °C; desolvation temperature 600 °C; desolvation gas flow (nitrogen) 800 L/h. AlkLactate was monitored with the MRM transitions m/z = 126.9 >83 (quantifier), and m/z = 126.9 >81 (qualifier) using a cone voltage of 25 V and collision energies of 8 (quantifier) and 12 eV (qualifier).

AlkLactate levels in unknown samples were derived from their peak area using a linear calibration model constructed from plotting the peak area versus concentration for the calibrator samples.

Synthesis

Analytical grade solvents and commercially available reagents were used without further purification unless otherwise noted. Methylglyoxal was prepared according to literature protocol.[ii] All reactions were carried out under regular atmosphere unless otherwise mentioned. Dry solvents were obtained from a MBraun MB SPS-800 solvent purification system or by drying over activated 4 Å molecular sieves overnight. For all reactions with inert reaction conditions glassware were flame dried under high vacuum followed by flushing with N_2. Reactions were monitored by TLC or ^{1}H NMR analysis. TLC was performed using pre-coated aluminum-backed plates (Merck TLC Silica gel 60 F254) and visualized by ultraviolet radiation, $KMnO_4$ or 2,4-dinitrophenylhydrazine stains. Flash chromatography (FC) was carried out on Fluka Analytical silica gel 60 (230-400 mesh). NMR spectra were acquired on a Bruker AVANCE III HD spectrometer running at 400 MHz for ^{1}H NMR and 100 MHz for ^{13}C NMR. Chemical shifts (δ) are reported in ppm relative to solvents residual resonance, ^{1}H NMR chemical shift: $CDCl_3$ (δ = 7.26), D_2O (δ = 4.79), CD_3OD (δ = 3.31), $(CD_3)_2SO$ (δ = 2.50), $(CD_3)_2CO$ (δ = 2.05) and CD_3CN (δ = 1.94). ^{13}C NMR chemical shifts: $CDCl_3$ (δ = 77.16), CD_3OD (δ = 49.00), $(CD_3)_2SO$ (39.52), CD_3COCD_3 (δ = 29.84), CD_3CN (δ = 1.32) and unadjusted when using D_2O as solvent. ^{1}H NMR spectra are reported as follows: chemical shift (coupling pattern where s = singlet, d = doublet, t = triplet, q = quartet, quin = quintet, hex = hextet, hep = heptet, oct = octet, m = multiplet, br = broad, coupling constant(s) in Hz, integration). ^{13}C NMR spectra are reported with chemical shift followed by (XC) for X identical carbons different than one. High resolution mass spectrometry (HRMS) spectra were recorded on a Bruker Maxis Impact mass spectrometer using ESI (referenced to the mass of the charged species).

Compound **9**

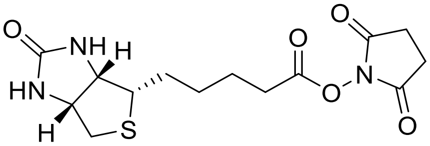

To a solution of biotin (1.98 g, 8.10 mmol) and *N*-hydroxysuccnimide (1.03 g, 8.91 mmol) in anhydrous DMF (60 mL) under inert atmosphere of N_2 was added dicyclohexylcarbodiimide (2.17 g, 10.54 mmol). The reaction was stirred for 44 h at rt The byproduct, dicyclohexylurea, was filtered off and washed with CH_2Cl_2. Solvents were removed *in vacuo*. First Et_2O (60 mL) was added to dissolve impurities and stirred for 15 min before filtering off the undissolved product, then 400 mL isopropanol was added and the reaction was heated to reflux and allowed to cool to rt before filtering off the desired product (2.47 g, 89%) as white powder. ^{1}H NMR (400 MHz, CD_3OD) δ (ppm): 4.50 (dd; *J* = 7.6, 4.6 Hz; 1H), 4.32 (dd; *J* = 7.6, 4.6 Hz; 1H), 3.22 (m, 1H), 2.93 (dd, *J* = 12.6, 4.9 Hz, 1H), 2.84 (s; 4H) 2.72 (d; *J* = 12.6 Hz; 1H), 2.66 (t, *J* = 7.2 Hz; 2H) 1.83-1.51 (m; 6H). ^{13}C NMR (100 MHz, CD_3OD) δ (ppm): 170.5 (2C), 168.8, 162.9, 61.9, 60.2, 55.4, 39.6, 30.0, 27.9, 25.1 (2C), 24.3, 24.2. HRMS (ESI)

m/z calcd for $C_{14}H_{20}N_3O_5S^+$ [M+H$^+$]: 342.1118, found: 342.1123. NMR data was in accordance with those published previously.[iii]

Compound **10**

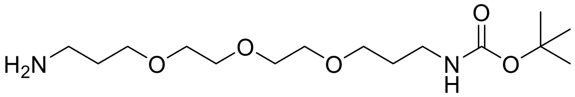

Di-*tert*-butyl bicarbonate (9.71 g, 44.03 mmol) dissolved in CH_2Cl_2 (90 mL) was added drop wise (over 2 h) to a solution of 4,7,10-trioca-1,13-tridecanamine (10 mL, 44.03 mmol) in CH_2Cl_2 (70 mL) with stirring and cooling in an ice bath. The ice bath was allowed to warm to rt overnight. The reaction mixture was concentrated *in vacuo* and dissolved in H_2O (300 mL). The solution was acidified to pH = 4 with 1 M HCl and washed with CH_2Cl_2 (3 x 150 mL). The aqueous phase was strongly basified with 2 M KOH (pH = 12-13) and extracted with CH_2Cl_2 (3 x 150 mL). The organic phases from the basified extraction were combined, dried over Na_2SO_4 and concentrated under vacuum, affording the product (4.01g, 28%) as a pale yellow oil. ^{1}H NMR (400 MHz, $CDCl_3$) δ (ppm): 5.13 (b s; 1H), 3.63-3.42 (m; 12H), 3.19-3.12 (m; 2H), 2.73 (t; J = 6.8; 2H), 1.72-1.60 (m; 4H), 1.37 (s; 9H), 1.25 (s; 2H). ^{13}C NMR (100 MHz, $CDCl_3$) δ (ppm): 156.0, 78.7, 70.6, 70.6, 70.2, 70.2, 69.5, 69.4, 39.6, 38.5, 33.3, 29.6, 28.4 (3C). HRMS (ESI) m/z calcd for $C_{15}H_{33}N_2O_5^+$ [M+H$^+$]: 321.2384, found: 321.2390. NMR data was in accordance with those published previously.[iv]

Compound **11**

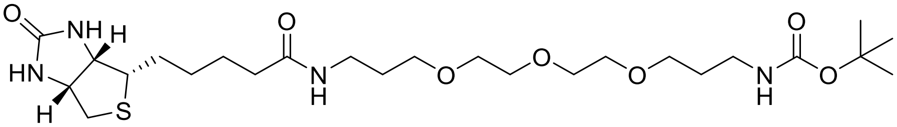

Under an inert atmosphere of N_2, compound **9** (450 mg, 1.32 mmol) and compound **10** (422 mg, 1.32 mmol) were dissolved in DMF (15 mL) and stirred at rt for 16 h. The reaction mixture was partitioned in CH_2Cl_2/H_2O (20 mL/25 mL) and the CH_2Cl_2 layer was separated. The aqueous layer was extracted four additional times with CH_2Cl_2 (17 mL) and all organic layers were combined and washed with saturated Na_2CO_3 (35 mL). The Na_2CO_3 layer was extracted with CH_2Cl_2 (25 mL) and CH_2Cl_2 layers were combined again, washed with brine (75 mL), dried over anhydrous $MgSO_4$, filtered and concentrated. DMF was removed under high vacuum at 60 °C and the resultant residue (orange sticky solid) was purified with FC using a 10-15% gradient of MeOH in CH_2Cl_2 to yield the desired product (420 mg, 58%) as a yellow sticky solid. ^{1}H NMR (400 MHz, $CDCl_3$) δ (ppm): 6.59 (s; 1H), 6.19 (s; 1H), 5.39 (s; 1H), 5.06 (s; 1H), 4.52 (m; 1H), 4.34 (m; 1H), 3.72-3.47 (m; 12H), 3.36 (q; *J* = 6.2 Hz; 2H), 3.23 (m; 3H), 2.92 (dd; *J* = 12.6, 4.9 Hz; 1H), 2.78 (d; *J* = 12.6 Hz; 1H), 2.21 (t; *J* = 7.5 Hz; 2H), 2.01-1.23 (m; 10H), 1.46 (s; 9H). ^{13}C NMR (100 MHz, $CDCl_3$) δ (ppm): 173.04, 163.64, 156.07, 78.98, 70.53, 70.49, 70.20, 70.10, 70.05, 69.52, 61.81, 60.14, 55.56, 40.56, 38.48, 37.87, 36.00, 29.71, 28.89, 28.48 (3C), 28.19, 28.12, 25.65. HRMS (ESI) m/z calcd for $C_{25}H_{47}N_4O_7S^+$ [M+H$^+$]: 547.3160, found: 547.3163. NMR data was in accordance with those published previously.[iv]

Compound **12**

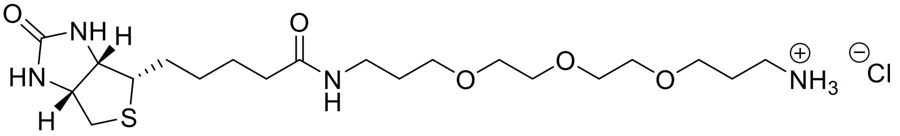

Anhydrous CH_2Cl_2 (8 mL) was added into a 50 mL round-bottomed flask equipped with a stir bar containing compound **11** (928 mg, 1.70 mmol) under inert atmosphere of N_2, forming a homogenous solution. The round-bottomed flask was cooled in an ice-water bath and TFA (8 mL) was added. The ice-water bath was removed and the reaction solution was stirred for 1 h. CH_2Cl_2 and TFA were removed *in vacuo*. 1 M HCl (4.5 mL) followed by H_2O (4.5 mL) was added to the concentrate to make it come into solution, stirred for 10 min and then concentrated again to yield the product as a chloride salt in quantitative yield as a sticky solid. ^{1}H NMR (400 MHz, D_2O) δ (ppm): 4.46 (m; 1H), 4.27 (m; 1H), 3.57-3.38 (m; 12H), 3.18 (quin; *J* = 4.6 Hz; 1H), 3.11 (t; *J* = 6.7 Hz; 2H), 2.96 (t; *J* = 7.0 Hz; 2H), 2.83 (dd; *J* = 4.9, 13.0 Hz; 1H), 2.64 (d; *J* = 13.0 Hz; 1H), 2.11 (t; *J* = 7.2 Hz; 2H), 1.80 (quin; *J* = 6.6 Hz; 2H), 1.50 (m; 8H). ^{13}C NMR (100 MHz, D_2O) δ (ppm): 176.7, 165.3, 69.5, 69.5, 69.4, 69.3, 68.3, 68.2, 62.1, 60.2, 55.3, 39.6, 37.6, 36.3, 35.4, 28.2, 27.8, 27.6, 26.4, 25.1. HRMS (ESI) m/z calcd for $C_{20}H_{39}N_4O_5S^+$ [M+H$^+$]: 447.2636, found: 447.2645. NMR data was in accordance with those published previously.[iv]

Compound **13**

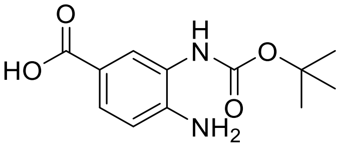

To a solution of di-*tert*-butyl dicarbonate (3.5 g, 15.9 mmol) in 25 mL dioxane:H_2O (1:1) was added 3,4-diaminobenzoic acid (1.0 g, 6.4 mmol) followed by Et_3N (2.7 mL, 19.1 mmol) and catalytic amount of DMAP (80 mg, 0.6 mmol). The reaction mixture was stirred at rt for 72 h then poured into 100 mL of H_2O. The pH was adjusted to 5 using 1 M HCl. The aqueous solution was extracted with EtOAc (3 x 125 mL). The combined organic layers were washed with H_2O (4x50 mL), dried over Na_2SO_4, filtered, and concentrated *in vacuo* to dryness. The crude product was recrystallized from MeOH (11 mL) to give the mono-Boc product as an off-white powder (530 mg, 33%). ^{1}H NMR (400 MHz, DMSO-d6) δ (ppm): 12.10 (s, 1H), 8.31 (s, 1H) 7.85 (s, 1H), 7.44 (d, *J* = 8.4 Hz, 1H), 6.67 (d, *J* = 8.4 Hz, 1H), 5.61 (s, 2H), 1.45 (s, 9H). NMR data was in accordance with those published previously.[v]

Probe **1**

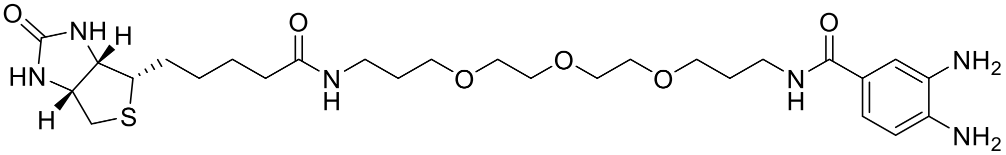

To a flame dried Schlenk flask purged with N_2, the carboxylic acid **13** (287 mg, 1.14 mmol) and HATU (487 mg, 1.24 mmol) were dissolved in anhydrous DMF followed by DIPEA (0.73 mL, 4.14 mmol) addition. The solution was stirred at rt for 5 min. Too this solution was added compound **12** (500 mg, 1.04 mmol) dissolved in

anhydrous DMF (5.5 mL). The solution was stirred at rt for 24 h before 2 M KOH (2.3 mL, 4.68 mmol) was added allowing removal of DIPEA under high vacuum overnight. The residue was dissolved in CH_2Cl_2 (6 mL) and TFA (6 mL) and stirred for 1 h at rt to allow complete removal of the Boc-protecting group then concentrated *in vacuo*. The residue was dissolved in H_2O (100 mL) and washed with CH_2Cl_2 (3 x 100 mL). The aqueous phase was concentrated *in vacuo* and the residue was purified with FC (10% MeOH in CH_2Cl_2) to yield the desired product (431 mg, 72%) as a yellow solid. ^1H NMR (400 MHz, D_2O) δ (ppm): 7.21 (d, *J* = 2.0 Hz, 1H), 7.19 (dd, *J* = 2.1, 8.1 Hz, 1H) 6.86 (d, *J* = 8.1 Hz, 1H), 4.55 (m, 1H), 4.34 (m, 1H), 3.68-3.58 (m, 10H), 3.53 (t, *J* = 6.4 Hz, 2H), 3.43 (t, *J* = 6.7 Hz, 2H), 3.26-3.19 (m, 3H), 2.94 (dd, *J* = 5.0, 13.1 Hz, 1H), 2.74 (d, *J* = 13.1 Hz, 1H), 2.20 (t, *J* = 7.3, 2H), 1.88 (quin, *J* = 6.5 Hz, 2H), 1.75 (quin, *J* = 6.5 Hz, 2H), 1.70-1.46 (m, 4H), 1.40-1.24 (m, 2H). ^{13}C NMR (100 MHz, D_2O) δ (ppm): 176.5, 170.2, 165.3, 138.9, 133.3, 124.6, 120.1, 116.1, 116.0, 69.6, 69.6, 69.4, 69.3, 68.8, 68.4, 62.0, 60.2, 55.3, 39.7, 37.2, 36.4, 35.5, 28.4, 28.2, 27.9, 27.6, 25.1. HRMS (ESI) m/z calcd for $C_{27}H_{45}N_6O_6S^+$ [M+H$^+$]: 581.3116, found: 581.3125.

Compound **14**

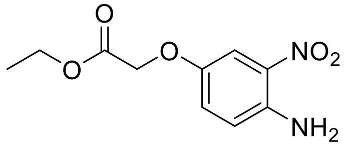

To a flame dried Schlenk flask charged with N_2, 4-amino-3-nitrophenol (2.00 g, 12.7 mmol) was dissolved in acetone (40 mL). K_2CO_3 (5.27 g, 38.2 mmol) and ethyl bromoacetate (2.55 g, 15.3 mmol) were added to the solution and stirred 18 h at rt. The reaction was concentrated *in vacuo* and H_2O (100 mL) and EtOAc (100 mL) were added, the layers were separated and the aqueous phase was extracted with EtOAc (3 x 100mL). The combined organic phases were washed with brine (100 mL) then dried with $MgSO_4$. The solvent was removed *in vacuo* yielding the product as a yellow solid without further purification necessary (2.79 g, 91%). HRMS (ESI) m/z calcd. For $C_{10}H_{13}N_2O_5^+$ [M+H$^+$]: 241.0819, found: 241.0821. NMR data was in accordance with those published previously.[vi]

Compound **15**

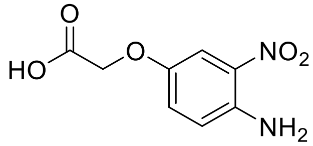

Compound **14** (2.21 g, 9.20 mmol) was added to MeOH (30 mL) and 2 M KOH (10 mL, 20 mmol). The reaction refluxed at 70 °C for 1 h. The solvent was evaporated *in vacuo*. The crude mixture was dissolved in 5 mL H_2O which was neutralized with 2 M HCl (~10 mL). Additional H_2O (85 mL) was added and the resulting aqueous solution was extracted with EtOAc (4 x 100 mL). The combined organic phases were washed with brine and dried over $MgSO_4$. The solution was concentrated *in vacuo* yielding the hydrolysis product as an orange solid (1.43 g, 73%). HRMS (ESI) m/z calcd. For $C_8H_9N_2O_5^+$ [M+H$^+$]: 213.0506, found: 213.0507. NMR data was in accordance with those published previously.[vi]

Compound **16**

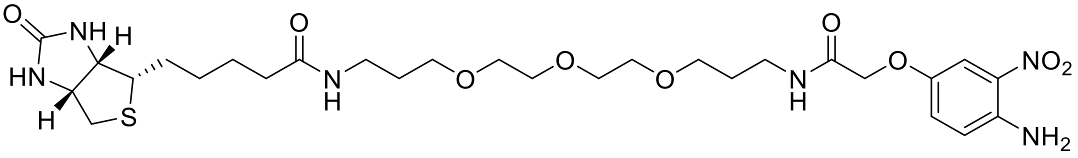

To a flame dried Schlenk flask purged with N_2, the carboxylic acid **15** (69 mg, 0.32 mmol) and HATU (137 mg, 0.35 mmol) were dissolved in anhydrous DMF (1.5 mL) and Et_3N (0.15 mL, 1.08 mmol) was added. The solution was stirred at rt for 5 min. Too this solution was added **12** (130 mg, 0.27 mmol) dissolved in anhydrous DMF (3 mL). The solution was stirred at rt for 24 h before 2 M KOH (0.7 mL, 1.4 mmol) was added allowing removal of Et_3N under reduced pressure. The residue was dissolved in H_2O (20 mL) and extracted with EtOAc (3 x 20 mL) and purified with FC (10% MeOH in CH_2Cl_2) yielding the product (35 mg, 20%). No representable NMR was acquired as the probe design, Probe **2**, was discontinued due to instability of Probe **2**. HRMS (ESI) m/z calcd. For $C_{28}H_{45}N_6O_9S^+$ [M+H$^+$]: 641.2963, found: 641.2968.

Probe **3**

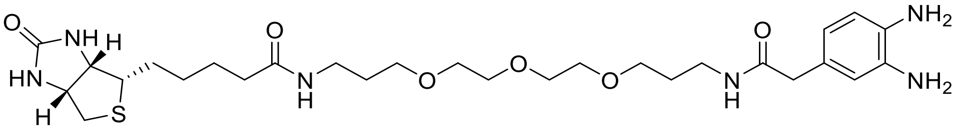

To a flame dried Schlenk flask was added 3,4-diaminophenylacetic acid (69 mg, 0.42 mmol), DCC (91 mg, 0.44 mmol) and NHS (54 mg, 0.46 mmol) under inert conditions. This was dissolved in DMF (0.5 mL) and immediately after Et_3N (0.17 mL, 1.25 mmol) was added and the mixture stirred at rt for 1 min. To this stirred solution was added **12** (200 mg, 0.42 mmol) in DMF (1.5 mL) via syringe. The solution was stirred at rt for 18 h. Precipitate was removed by filtration and KOH (0.48 mmol) was added allowing Et_3N to be removed *in vacuo*. The mixture was concentrated *in vacuo*, prior to purification with FC using MeOH/CH_2Cl_2 as eluent (gradient of 1:12 to 1:6) affording the product (120 mg, 49%) as an yellow solid. ^{1}H NMR (400 MHz, D_2O): δ 6.77 (d, *J*=2.9 Hz, 1H), 6.72 (s, 1H), 6.66 (d, *J*=2.9 Hz, 1H), 4.58-4.52 (m, 1H), 4.37-4.32 (m, 1H), 3.68-3.45 (m, 12H), 3.38 (s, 2H), 3.29-3.18 (m, 5H), 2.93 (dd, *J*=13.0; 4.7 Hz, 1H), 2.73 (d, *J*=13.0 Hz, 1H), 2.20 (t, *J*=7.0 Hz, 2H), 1.79-1.47 (m, 8H), 1.40-1.30 (m, 2H). ^{13}C NMR (100 MHz, D_2O): δ 176.6, 174.9, 165.3, 134.5, 133.0, 127.3, 121.1, 117.9, 117.8, 69.52, 69.49, 69.34, 69.30, 68.4, 68.3, 62.0, 60.2, 55.3, 41.9, 39.6, 36.5, 36.3, 35.5, 28.2, 28.1, 27.9, 27.6, 25.1. HRMS (ESI+) *m/z* calcd for $C_{28}H_{47}N_6O_6S^+$ [M+H]$^+$: 595.3272, found: 595.3273.

Product **6**

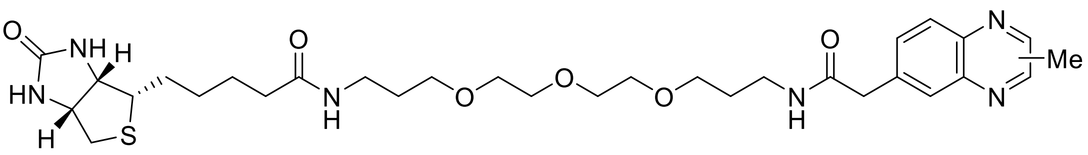

Regioisomeric ratio 1:1

Aqueous solution of MG (60 mM, 2.4 mL, 0.14 mmol) was added to a solution of Probe **3** (78 mg, 0.13 mmol) in H_2O (3 mL) and stirred for 30 min at rt. The mixture was then diluted to 25 mL with H_2O and washed with EtOAc (1 x 25mL). The aqueous phase was then carefully extracted multiple times with CH_2Cl_2 (9 x 50mL). The

combined organic phases were dried using $MgSO_4$ and concentrated *in vacuo* prior to purification with FC using MeOH/CH_2Cl_2 as eluent (1:10) affording the product (40 mg, 48%) as an off-white solid. The ratio of regioisomers was 1:1. Characterization is given as a mix of the two regioisomers. ^{1}H NMR (400 MHz, D_2O): δ 8.56 (s, ½H), 8.54 (s, ½H), 7.82-7.57 (m, 3H), 4.52-4.44 (m, 1H), 4.28-4.22 (m, 1H), 3.73 (s, 2H), 3.55-3.43 (m, 12H), 3.26 (t, *J*=6.5 Hz, 2H), 3.17-3.09 (m, 3H), 2.86 (dd, *J*=13.1; 4.7 Hz, 1H), 2.67 (d, *J*=13.1 Hz, 1H), 2.61 (s, 3H), 2.10 (t, *J*=7.0 Hz, 2H), 1.75 (quin, *J*=6.3 Hz, 2H), 1.68 (quin, *J*=6.3Hz, 2H), 1.62-1.35 (m, 4H), 1.28-1.16 (m, 2H). ^{13}C NMR (100 MHz, D_2O): δ 176.3, 173.2 (½C), 173.1 (½C), 165.1, 154.9 (½C), 154.5 (½C), 146.3 (½C), 145.9 (½C), 140.3 (½C), 139.5 (½C), 139.2 (½C), 138.3 (½C), 138.3 (½C), 137.1 (½C), 131.9 (½C), 130.8 (½C), 128.0 (½C), 127.4 (½C), 127.3 (½C), 126.8 (½C), 69.5, 69.4, 69.3 (2C), 68.4, 68.3, 61.9, 60.1, 55.3, 42.5 (½C), 42.4 (½C), 39.6, 36.6, 36.3, 35.4, 28.2, 27.8, 27.6, 25.1, 21.23, 21.16. HRMS (ESI+) *m/z* calcd. for $C_{31}H_{47}N_6O_6S^+$ $[M+H]^+$: 631.3272, found: 631.3277.

Product **7**

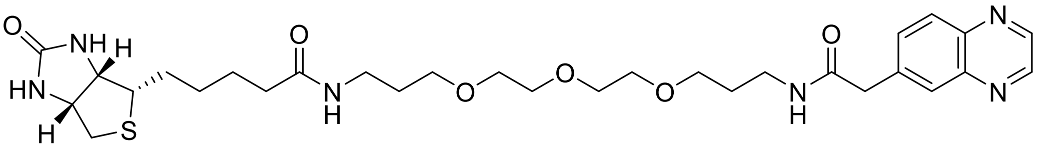

Aqueous solution of glyoxal (4.2 mg, 72 µmol) was added to a solution of Probe **3** (43 mg, 72 µmol) in H_2O (3 mL) and stirred for 20 min at rt. The mixture was then diluted to 25 mL with H_2O and washed with EtOAc (1 x 25mL). The aqueous phase was then carefully extracted multiple times with CH_2Cl_2 (15 x 25mL). The combined organic phases were dried using $MgSO_4$ and then concentrated *in vacuo* yielding the product (30 mg, 67%) as an off-white solid without further purification needed. ^{1}H NMR (400 MHz, D_2O): δ 8.80 (s, 1H), 8.79 (s, 1H), 7.96 (d, *J*=8.6 Hz, 1H), 7.87 (s, 1H), 7.74 (d, *J*=8.6 Hz, 1H), 4.53-4.46 (m, 1H), 4.30-4.25 (m, 1H), 3.79 (s, 2H), 3.57-3.45 (m, 12H), 3.27 (t, *J*=6.5 Hz, 2H), 3.19-3.12 (m, 3H), 2.88 (dd, *J*=13.1; 4.7 Hz, 1H), 2.68 (d, *J*=13.1 Hz, 1H), 2.12 (t, *J*=7.0Hz, 2H), 1.76 (quin, *J*=6.3 Hz, 2H), 1.69 (quin, *J*=6.3 Hz, 2H), 1.63-1.37 (m, 4H), 1.30-1.19 (m, 2H). ^{13}C NMR (100 MHz, D_2O): δ 176.4, 173.1, 165.2, 145.3, 144.9, 141.4, 140.6, 138.5, 132.1, 128.4, 127.8, 69.5, 69.4, 69.3 (2C), 68.4, 68.3, 62.0, 60.1, 55.3, 42.4, 39.6, 36.6, 36.2, 35.4, 28.2, 28.1, 27.8, 27.6, 25.1. HRMS (ESI+) *m/z* calcd. for $C_{30}H_{45}N_6O_6S^+$ $[M+H^+]$: 617.3116, found: 617.3120.

Compound **17**

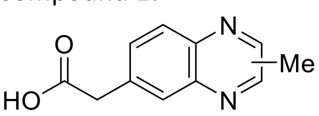

Regioisomeric ratio 1:1

3,4-Diaminophenylacetic acid (102 mg, 0.61 mmol) was dissolved in a aqueous TCA solution (3 wt.%, 10 mL) before 60 mM MG solution (1.1 mL, 0.67 mmol) was added. The solution was allowed to stir at rt for 1 h before being extracted with CH_2Cl_2 (4 x 10 mL). The combined organic phase was dried over $MgSO_4$ before concentration *in vacuo*. The residue was purified with FC (2% MeOH and 0.5% formic acid in CH_2Cl_2) yielding the product as an orange/brown solid (111 mg, 90%). The obtained ratio of regioisomers was 1:1 under the applied conditions. Characterization is given as a mix of the two. ^{1}H NMR (400 MHz, $CDCl_3$): δ 12.54 (s, br, 1), 8.78 (2×s, 1H), 8.08-8.03 (m, 2 H), 7.75-7.68 (2×d, *J* = 8.7/8.6 Hz, 1H), 3.91 (s, 2 H), 2.79 (s, 3 H). ^{13}C NMR (100 MHz, CDCl3): δ 175.3, 175.2, 154.3, 154.1, 145.7, 145.6, 141.4, 140.9, 140.0, 139.5, 137.0, 135.8, 132.6, 131.5,

128.8, 128.7, 128.4, 128.3, 41.4, 41.2, 22.3, 22.2. HRMS (ESI) m/z calcd. for $C_{11}H_{11}N_2O_2^+$ [M+H$^+$]: 203.0815, found: 203.0815.

Compound **18**

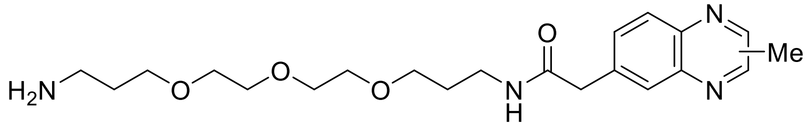

To a flame dried Schlenk flask with N_2 was added **17** (115 mg, 0.57 mmol), DCC (119 mg, 0.57 mmol) and NHS (70 mg, 0.60 mmol). This was dissolved in DMF (2 mL) and immediately after Et_3N (151 µL, 1.08 mmol) was added and the mixture stirred at rt for 3 min. To this stirred solution was added compound **10** (174 mg, 0.54 mmol) in DMF (2 mL) via syringe. The solution was stirred at rt for 18 h. The precipitate was removed by filtration and solvent removed *in vacuo*. The residue was dissolved in H_2O (50 mL) and extracted with CH_2Cl_2 (9 x 50 mL CH_2Cl_2). The combined organic phases were dried using $MgSO_4$ and concentrated *in vacuo*. The crude was dissolved in CH_2Cl_2 (2 mL) and transferred to a round bottomed flask equipped with stirring bar. To this solution was added TFA (2 mL) and the solution was stirred for 16 h. The solvents was removed under reduced pressure and the residue then dissolved in aqueous NaOH (0.05 M, 25 mL) and extracted with CH_2Cl_2 (15 x 25 mL). The product was finally purified with FC using a 10-70% gradient of MeOH in CH_2Cl_2 containing 0.5% NH_4OH. This yielded the desired product (60 mg, 27% yield) as a slightly yellow solid. The ratio of regioisomers was 1:1. Characterization is given as a mix of the two. ^{1}H NMR (400 MHz, $CDCl_3$) δ (ppm): 2.72 (2×s, 1H), 8.00 (2×d, *J* = 8.6 Hz, 1H), 7.91 (2×d, *J* = 1.5 Hz, 1H), (2×dd, *J* = 8.6; *J* = 1.5 Hz, 1H), 6.72 (s, 1H), 3.74 (s, 2H), 3.60-3.34 (m, 14H), 2.78-2.72 (m, 5H), 1.77-1.65 (m, 4H). ^{13}C NMR (100 MHz, $CDCl_3$) δ (ppm): 170.1, 154.1, 153.7, 146.2, 145.9, 142.1, 141.2, 141.0, 140.1, 137.9, 136.8, 131.7, 130.6, 129.5, 129.1, 129.0, 128.7, 70.5, 70.4, 70.1, 70.0, 69.5, 43.8, 43.7, 39.7, 38.3, 33.3, 28.7, 22.6, 22.6. HRMS (ESI) m/z calcd. for $C_8H_{11}N_2O_3^+$ [M+H$^+$]: 405.2496, found 405.2500.

NMR

Probe **1**

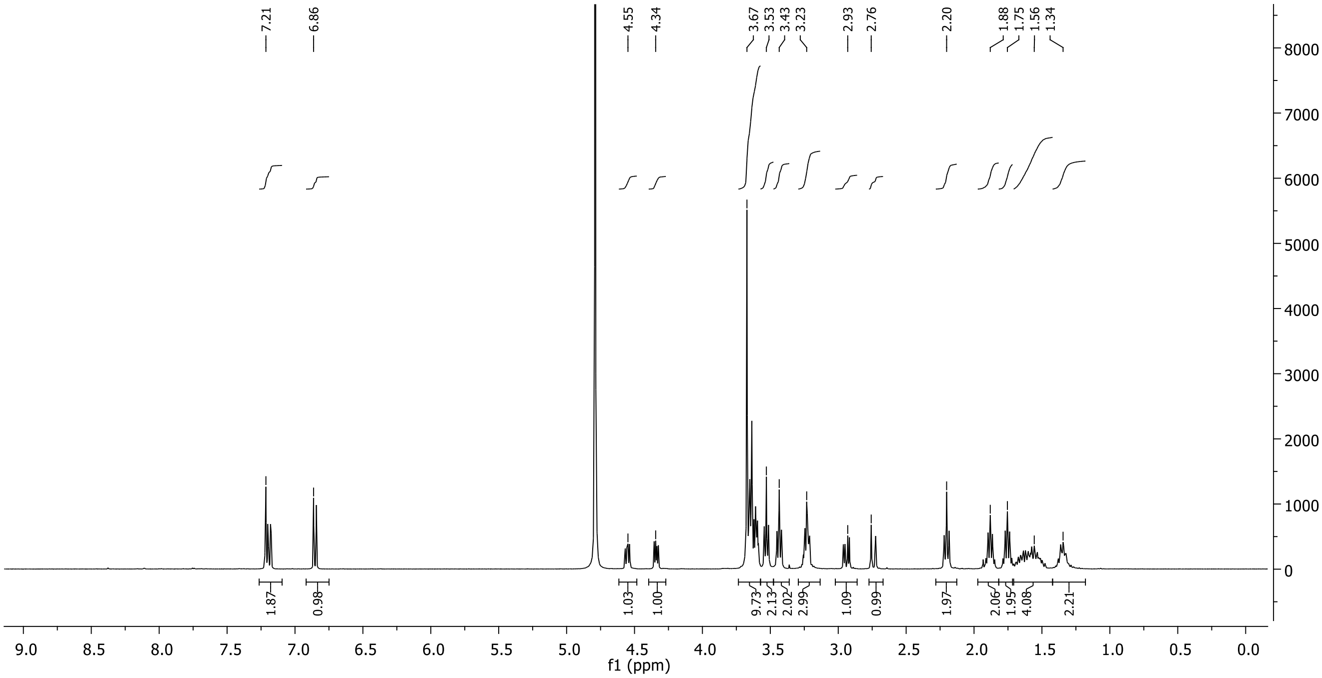

7.21
6.86
4.55
4.34
3.67
3.53
3.43
3.23
2.93
2.76
2.20
1.88
1.75
1.56
1.34
1.87
0.98
1.03
1.00
9.73
2.13
2.02
2.99
1.09
0.99
1.97
2.06
1.95
4.08
2.21
f1 (ppm)

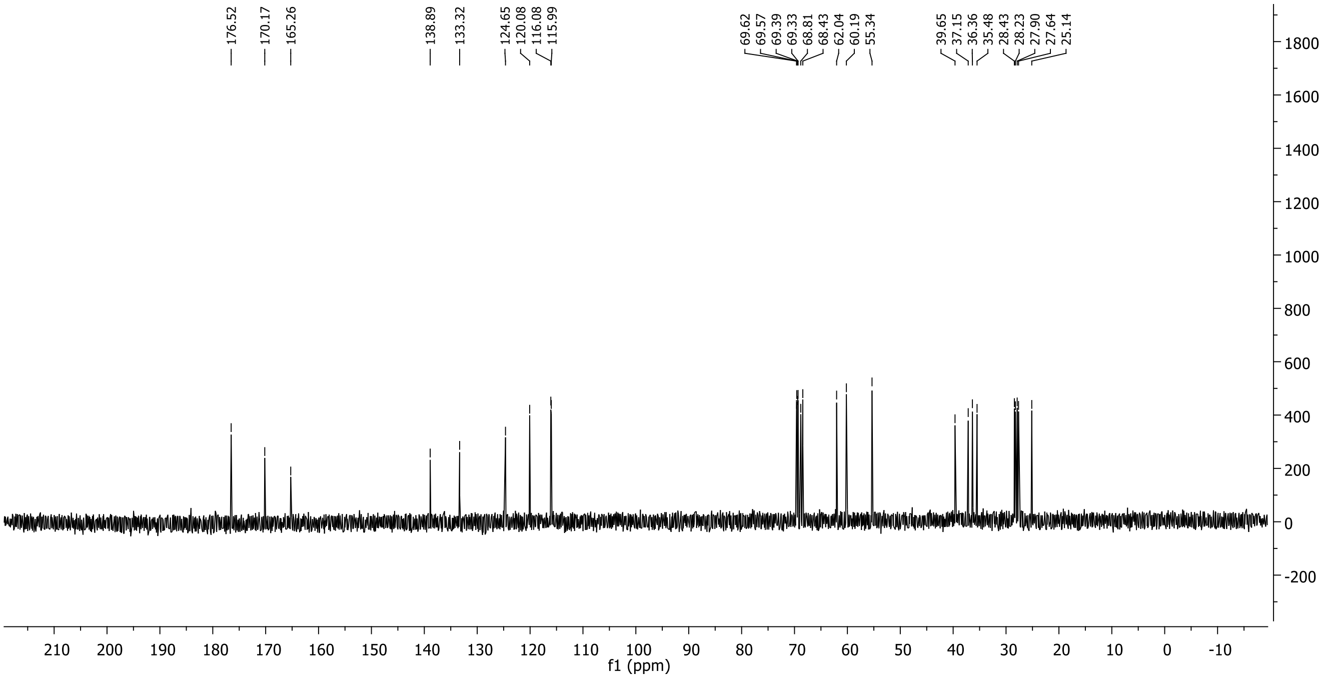

176.52
170.17
165.26
138.89
133.32
124.65
120.08
116.08
115.99
69.62
69.57
69.39
69.33
68.81
68.43
62.04
60.19
55.34
39.65
37.15
36.36
35.48
28.43
28.23
27.90
27.64
25.14
f1 (ppm)

Probe **3**

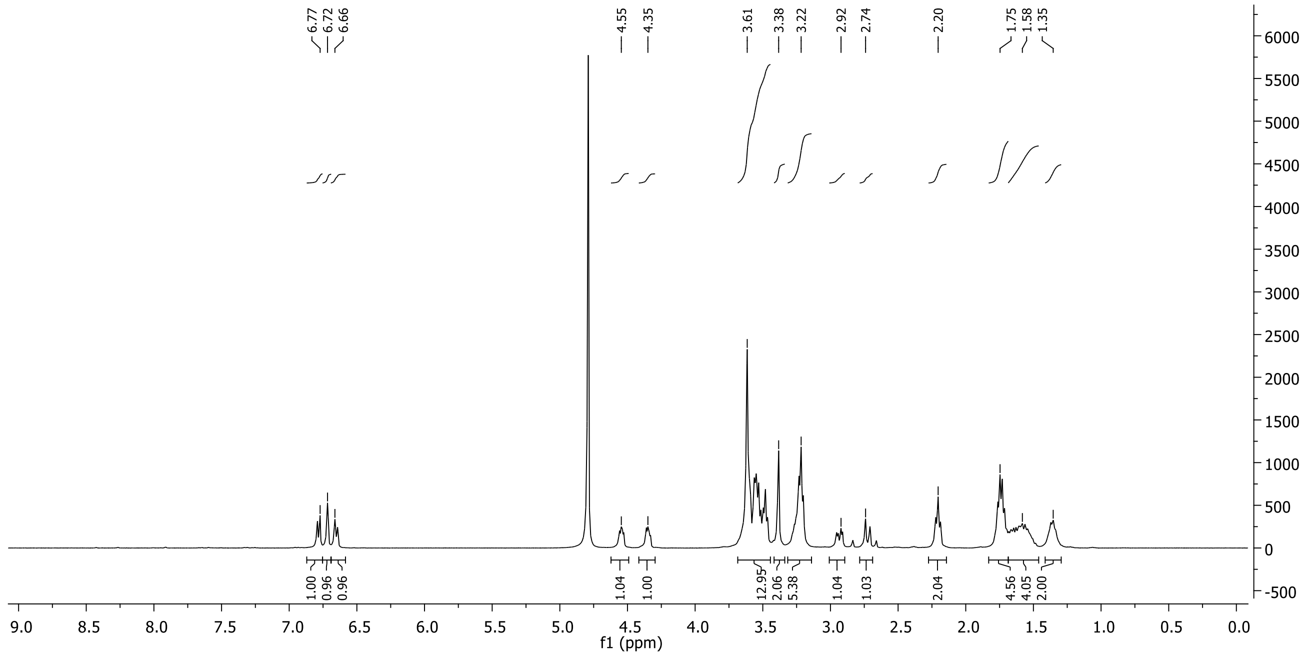

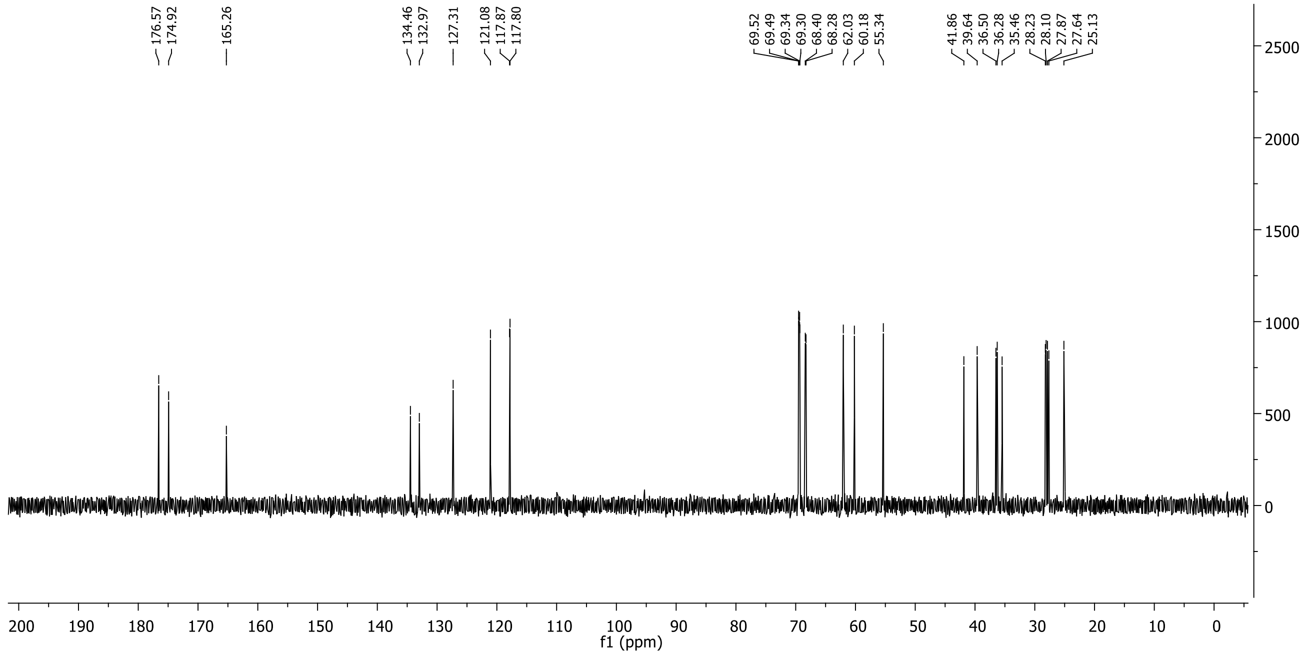

Product **6**

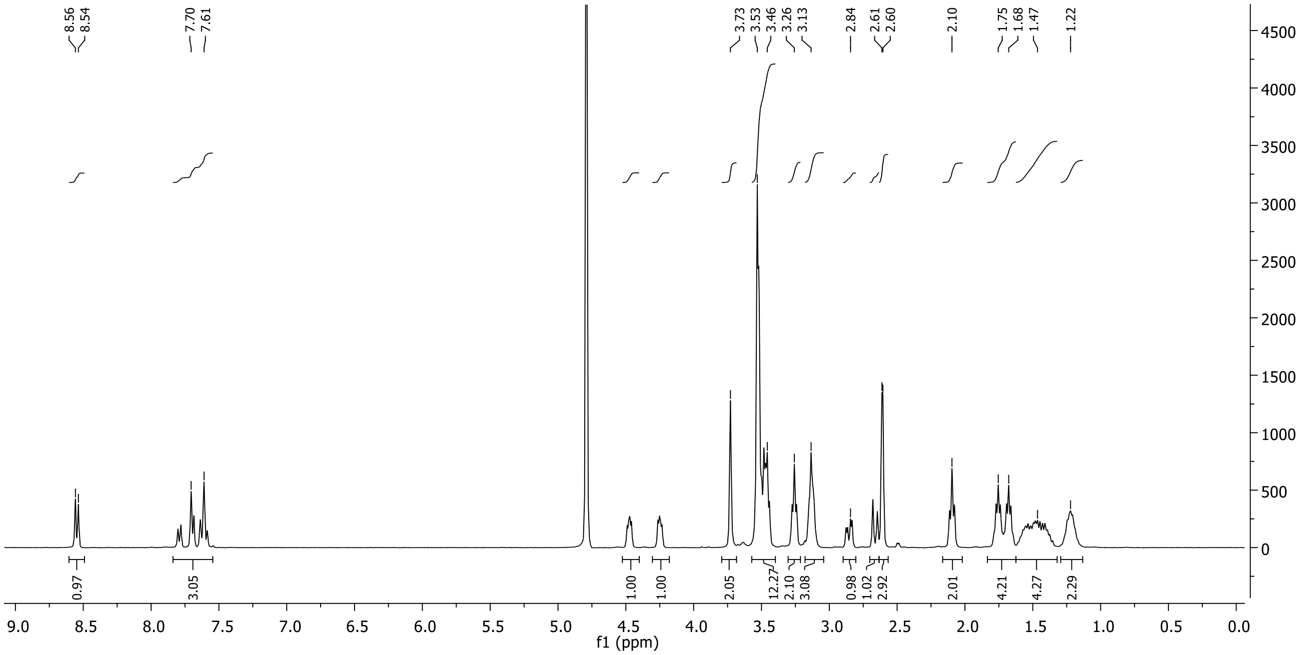
8.56
8.54
7.70
7.61
3.73
3.53
3.46
3.26
3.13
2.84
2.61
2.60
2.10
1.75
1.68
1.47
1.22
0.97
3.05
1.00
1.00
2.05
12.27
2.10
3.08
0.98
1.02
2.92
2.01
4.21
4.27
2.29
f1 (ppm)

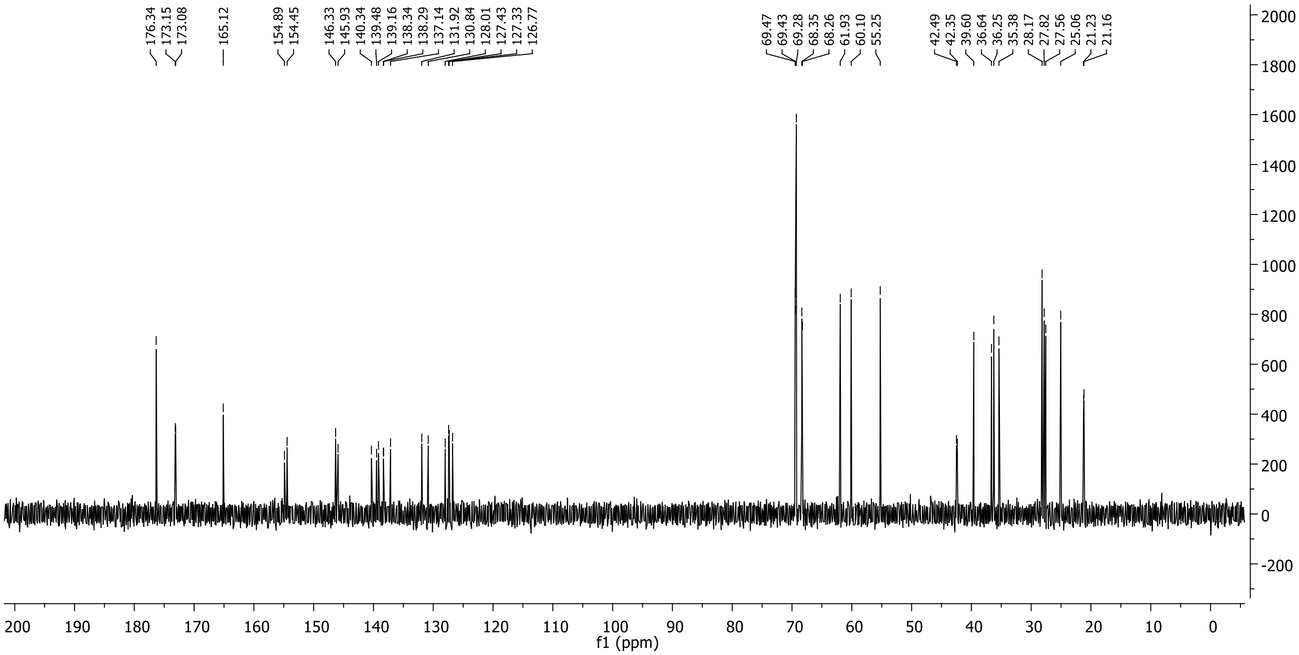
176.34
173.15
173.08
165.12
154.89
154.45
146.33
145.93
140.34
139.48
139.16
138.34
138.29
137.14
131.92
130.84
128.01
127.43
127.33
126.77
69.47
69.43
69.28
68.35
68.26
61.93
60.10
55.25
42.49
42.35
39.60
36.64
36.25
35.38
28.17
27.82
27.56
25.06
21.23
21.16
f1 (ppm)

Product **7**

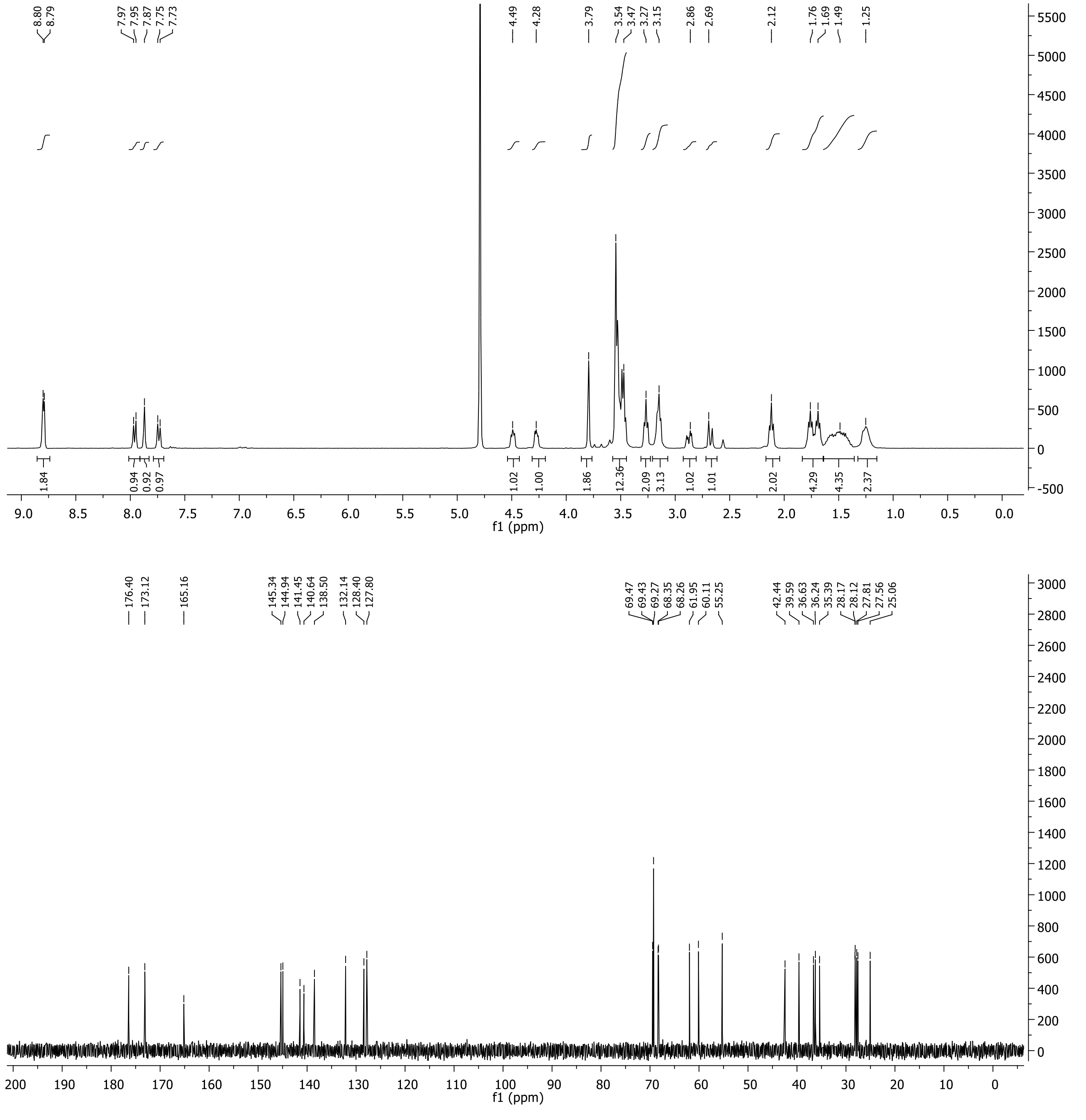
8.80
8.79
7.97
7.95
7.87
7.75
7.73
4.49
4.28
3.79
3.54
3.47
3.27
3.15
2.86
2.69
2.12
1.76
1.69
1.49
1.25
1.84
0.94
0.92
0.97
1.02
1.00
1.86
12.36
2.09
3.13
1.02
1.01
2.02
4.29
4.35
2.37
f1 (ppm)
176.40
173.12
165.16
145.34
144.94
141.45
140.64
138.50
132.14
128.40
127.80
69.47
69.43
69.27
68.35
68.26
61.95
60.11
55.25
42.44
39.59
36.63
36.24
35.39
28.17
28.12
27.81
27.56
25.06
f1 (ppm)

Compound **18**

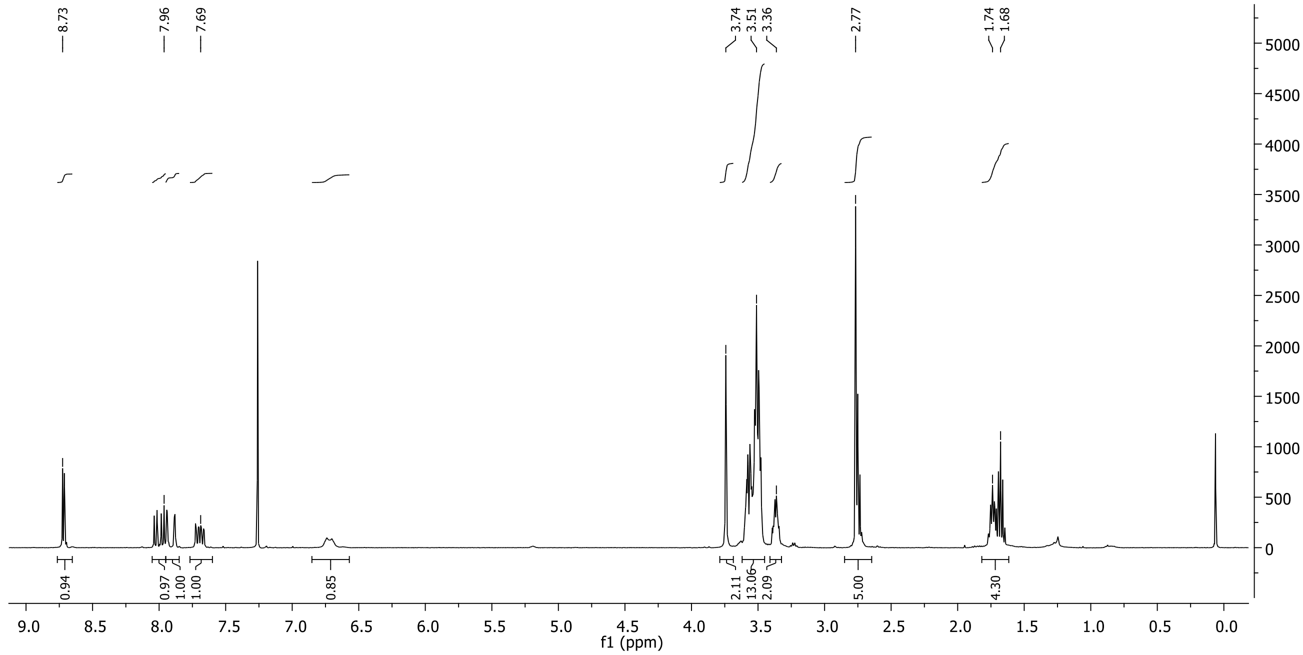

8.73
7.96
7.69
3.74
3.51
3.36
2.77
1.74
1.68
0.94
0.97
1.00
1.00
0.85
2.11
13.06
2.09
5.00
4.30
f1 (ppm)

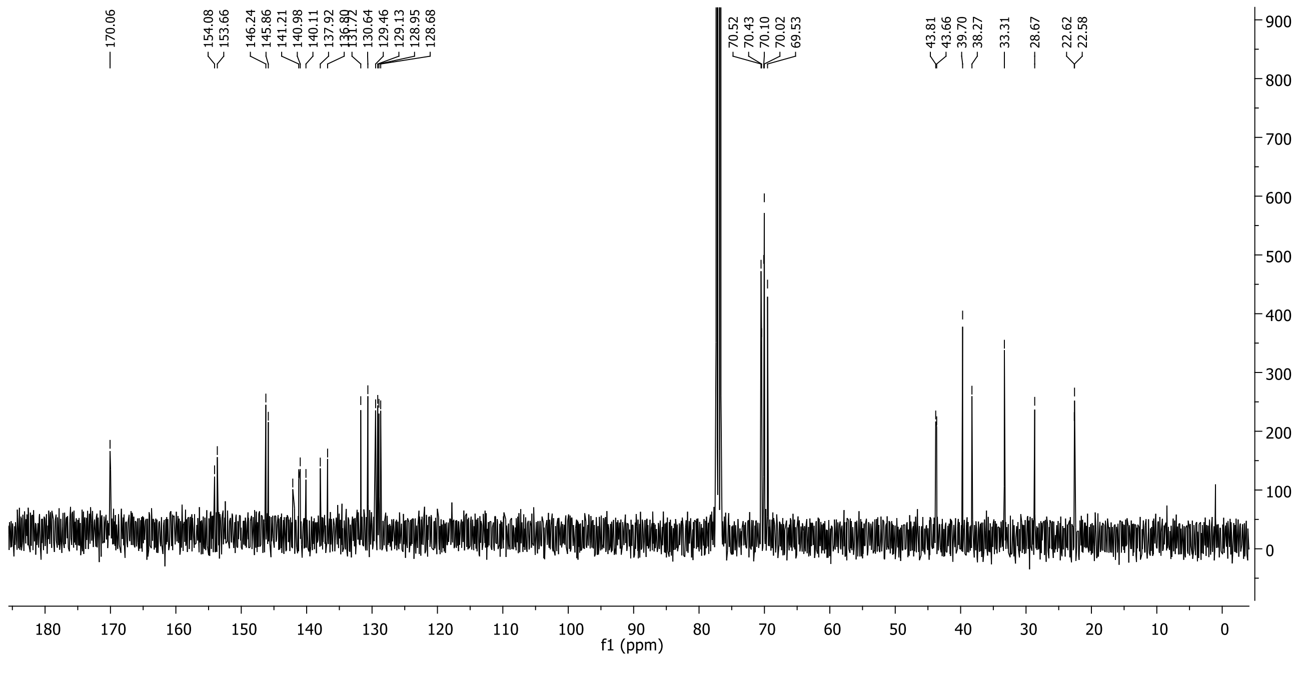

170.06
154.08
153.66
146.24
145.86
141.21
140.98
140.11
137.92
136.80
131.72
130.64
129.46
129.13
128.95
128.68
70.52
70.43
70.10
70.02
69.53
43.81
43.66
39.70
38.27
33.31
28.67
22.62
22.58
f1 (ppm)

References

[i]Bielefeld-Sevigny, M. *Assay Drug Dev Technol* **2009**, *7*, 90–92.

[ii]Rabbani, N.; Thornalley, P. J. *Nat Protoc* **2014**, *9*, 1969–1979.

[iii]Charvet, N.; Reiss, P.; Roget, A.; Dupuis, A.; Grünwald, D.; Carayon, S.; Chandezon, F.; Livache, T. *J. Mater. Chem.* **2004**, *14*, 2638–2642.

[iv]Chen, X.; Henschke, L.; Wu, Q.; Muthoosamy, K.; Neumann, B.; Weil, T. *Org. Biomol. Chem.* **2013**, *11*, 353–361.

[v]Jahani, F.; Tajbakhsh, M.; Golchoubian, H.; Khaksar, S. *Tetrahedron Lett.* **2011**, *52*, 1260–1264.

[vi] Lu, J.; Yang, H.; Jin, Y.; Jiang, Y.; Fu, H. *Green Chem.* **2013**, *15*, 3184–3187.
